# CircFam190a: a critical positive regulator of osteoclast differentiation via enhancement of the AKT1/HSP90β complex

**DOI:** 10.1038/s12276-023-01085-y

**Published:** 2023-09-01

**Authors:** Kun Chen, Xi Chen, Chuandong Lang, Xingshi Yuan, Junming Huang, Zhi Li, Mingyou Xu, Kerong Wu, Chenhe Zhou, Qidong Li, Chen Zhu, Lianxin Liu, Xifu Shang

**Affiliations:** 1 Department of Orthopedics, The First Affiliated Hospital of USTC, Division of Life Sciences and Medicine, University of Science and Technology of China, 230001 Hefei, Anhui China; 2 Department of Hepatobiliary Surgery, The First Affiliated Hospital of USTC, Division of Life Sciences and Medicine, University of Science and Technology of China, 230001 Hefei, Anhui China; 3 Department of Orthopedics, The First Affiliated Hospital of Nanchang University, 330000 Nanchang, Jiangxi China; 4 Department of Orthopedic Surgery, the Second Affiliated Hospital, Zhejiang University School of Medicine, 310009 Hangzhou, Zhejiang China; 5 Anhui Province Key Laboratory of Hepatopancreatobiliary Surgery, 230001 Hefei, Anhui China; 6 Anhui Provincial Clinical Research Center for Hepatobiliary Diseases, 230001 Hefei, Anhui China

**Keywords:** Targeted bone remodelling, Osteoporosis

## Abstract

The identification of key regulatory factors that control osteoclastogenesis is important. Accumulating evidence indicates that circular RNAs (circRNAs) are discrete functional entities. However, the complexities of circRNA expression as well as the extent of their regulatory functions during osteoclastogenesis have yet to be revealed. Here, based on circular RNA sequencing data, we identified a circular RNA, circFam190a, as a critical regulator of osteoclast differentiation and function. During osteoclastogenesis, circFam190a is significantly upregulated. In vitro, circFam190a enhanced osteoclast formation and function. In vivo, overexpression of circFam190a induced significant bone loss, while knockdown of circFam190a prevented pathological bone loss in an ovariectomized (OVX) mouse osteoporosis model. Mechanistically, our data suggest that circFam90a enhances the binding of AKT1 and HSP90β, promoting AKT1 stability. Altogether, our findings highlight the critical role of circFam190a as a positive regulator of osteoclastogenesis, and targeting circFam190a might be a promising therapeutic strategy for treating pathological bone loss.

## Bone disease: Circular RNA in bone formation

A newly discovered circular RNA molecule that promotes development of bone-degrading osteoclast cells offers a promising target for preventing skeletal degeneration in conditions like osteoporosis. Osteoclasts play a critical role in maintaining healthy bone density but excess activity can lead to eroded and fragile bone tissue. Several studies have suggested that gene-regulating circular RNAs play a role in osteoclast development, and Kun Chen of the University of Science and Technology of China, Hefei, and colleagues have identified one such molecule called circFam190a. They demonstrate that targeted inhibition of circFam190a can preserve bone integrity in a mouse model of osteoporosis without interfering with the activity of bone-generating osteoblast cells. The authors were also able to identify the broader network of signaling pathways influenced by circFam190a, which include several proteins with well-established roles in osteoclast function.

## Introduction

Osteoclasts play a central role in bone metabolism^1–4^. The regulation of osteoclasts is often disrupted under pathological conditions: excessive osteoclastogenesis is involved in various bone loss diseases, including osteoporosis, autoimmune arthritis, and bone metastasis, while defective osteoclastic function leads to osteopetrosis^5^. Therefore, identifying the key regulatory factors responsible for osteoclast differentiation is crucial for understanding the physiology and pathology of the skeletal system.

Circular RNAs (circRNAs), a family of noncoding RNAs, are generated from backsplicing of pre-mRNAs to form covalently closed transcripts^6^. Initially deemed erroneous byproducts of splicing, circular RNAs (circRNAs) are now known to be distinct functional entities that critically affect various biological processes and human diseases. These processes encompass fundamental aspects such as development, aging, and differentiation^7,8^. Notably, several recent studies have linked circRNAs to bone metabolism^9–11^: Zhai et al. reported that hsa_circ_0008500 could promote osteoblast mineralization by acting as a miR-1301-3p sponge^9^; Li’s study indicated that circCDR1as could regulate osteoblastic differentiation via the miR-7/GDF5/SMAD and p38 MAPK signaling pathways^10^. However, the complexities of circRNA expression as well as the extent of their regulatory functions during osteoclastogenesis have yet to be revealed.

It has been established that the serine/threonine kinase AKT, also known as protein kinase B, plays a crucial role in maintaining bone homeostasis^12^. AKT1 knockout mice showed an osteopenic phenotype due to dysfunction of osteoblasts and osteoclasts^13^. In detail, AKT1 is involved in osteoclast differentiation and survival as a part of the RANKL/NFKB axis^14,15^. Additionally, AKT has been shown to mediate the TGFβ1-dependent early phase of osteoblast differentiation^16,17^. Nevertheless, whether AKT is regulated by circular RNAs during osteoclast differentiation remains unknown.

For the current study, we aimed to discover the roles played by circRNAs during osteoclastogenesis. Our screening strategy identified a circRNA derived from Fam190a pre-mRNA, which we named circFam190a. The expression of circFam190a is dramatically increased during osteoclast differentiation. Both in vitro and in vivo studies suggest that circFam190a promotes osteoclast formation and function. Mechanistically, we show that circFam90a enhances the binding of AKT1 and HSP90β, thereby activating the AKT1 signaling pathway. Overall, our findings establish circFam190a as a critical positive regulator of osteoclast differentiation and function.

## Materials and methods

### Mouse studies

All animal experiments were approved by the Animal Care and Use Committee of the First Affiliated Hospital of USTC (ethical approval no. 2022-N(A)-078). All the procedures followed the guidelines set forth by the US National Institutes of Health (NIH Publication No. 85-23, revised 1996) on the protection of animals used for scientific purposes. Wild-type C57/BL6 mice were purchased from the Shanghai Slake Experimental Animal Company (Shanghai, China). All mouse strains were on a pure C57BL/6 genetic background and were housed in the animal facility of the Fist Affiliated Hospital of USTC in a pathogen-free, temperature-controlled environment under a 12:12 h light–dark cycle.

### Bone marrow macrophage (BMM) isolation

BMMs were isolated from 6-week-old mice as previously described^18^. Briefly, bone marrow cells were flushed from the femur and tibia and then resuspended in red blood lysis buffer (Beyotime, C3702-120 ml) for 2 min to remove red blood cells. The cells were cultured in complete α-MEM (Gibco, 22561-021) with 30 ng/ml M-CSF (R&D Systems, 416-ML-050) in a suspension culture dish (Corning, 430591) at 37 °C for 3 days. After that, nonadherent cells were removed by washing, and the attached BMMs were released using 0.25% trypsin-EDTA (Thermo Fisher, 25200056) for the indicated experiments.

### Osteoclast differentiation

For induction of osteoclast differentiation, BMMs were seeded in plates at a density of 25,000 cells/cm^2^. The cells were cultured in complete α-MEM with 30 ng/ml M-CSF and 10 ng/ml RANKL (R&D Systems, 462-TEC-010) for 5 days to obtain mature osteoclasts as previously described^18,19^. For circular RNA sequencing, BMMs were treated with RANKL and collected at the indicated time points corresponding to different osteoclast differentiation stages: D0, undifferentiated (without RANKL treatment); D1, early-stage (with RANKL treatment for 1 day); D3, middle-stage (with RANKL treatment for 3 days); and D5, late-stage (with RANKL treatment for 5 days).

### Circular RNA sequencing

For circular RNA sequencing, total RNA was extracted from BMMs treated with RANKL for the indicated time points (D0, D1, D3, D5) using TRIzol reagent (Invitrogen Thermo Fisher, 15596026) following the manufacturer’s instructions. The quantity and quality of the RNA were evaluated using a NanoDrop spectrophotometer and an Agilent 2100 bioanalyzer. Subsequently, DNase I was used to degrade double-stranded and single-stranded DNA. The Ribo-of rRNA Depletion kit (Vazyme Biotech Co., N406) was used to remove ribosomal RNA, and RNase R (Epicentre Illumina) was utilized to eliminate linear RNA. Purification was carried out using Agencourt RNAClean XP magnetic beads (Invitrogen; Thermo Fisher Scientific, Inc.). The libraries were sequenced on the BGISEQ-500 (BGI Group). Raw reads were filtered to remove those with low quality, linker contamination and excessively high levels of unknown base N using SOAPnuke software v1.5.2 (https://github.com/BGI-flexlab/SOAPnuke). The remaining clean reads were aligned to the reference genome (Mus_musculus, UCSC_mm9; ftp://hgdownload.soe.ucsc.edu/goldenPath/mm9/). Circular RNAs were detected and identified using CIRI^20^ and Find_circ^6,21^. The results from those two software tools were integrated based on the circRNA start and stop positions (combining circRNAs with start and stop positions within the first and last 10 bases into one class). The expression of circRNAs was calculated based on the number of junction reads aligned to the two ends of the circRNA. As both CIRI (BWA‑MEM genome alignment algorithm) and Find_circ (Bowtie2 genome alignment algorithm) software were used in the profiling, the final numbers of junction reads were the mean of the two results. CircRNAs exhibiting fold changes ≥2.0 with *P* values ≤0.05 were classified as significantly differentially expressed circRNAs. The differentially expressed genes (DEGs) obtained from all groups were analyzed by bidirectional clustering using the Pheatmap package (v1.0.12; rdocumentation.org/packages/pheatmap/versions/1.0.12) and are presented as heatmaps.

### Transcriptome analysis

For transcriptome RNA-seq analysis, BMMs transfected with ASO-ctrl or ASO-1 were cultured in the presence of M-CSF and RANKL for 5 days. Afterward, cells were collected, and total RNA was isolated using TRIzol reagent. RNA sequencing was performed by Novogene Company (Beijing, China). Subsequent data analysis was conducted by Rstudio and GSEA software.

### RNA preparation, RNase R treatment, qRT‒PCR and actinomycin D treatment

Total RNA was isolated from cells or tissues using TRIzol reagent according to the manufacturer’s instructions. For verification of the backspliced junction point of circRNAs, rRNA and linear RNA were removed using the rRNA Depletion kit (Vazyme Biotech Co., N406) and RNase R (Epicentre Technologies, Madison, WI). cDNA was synthesized using a random primer (TaKaRa, Dalian, China). For quantification of the mRNA and circRNA levels, cDNA was synthesized using PrimeScript RT Master Mix (TaKaRa, Dalian, China). Real-time PCR analyses were performed using SYBR Premix Ex Taq II (TaKaRa). Notably, divergent primers annealing at the distal ends of circRNA were used to determine the abundance of circRNA. Amplification was performed using the StepOnePlus Real-Time PCR System (Applied Biosystems, Foster City, CA), and Ct thresholds were determined using the software. All the primers used in this study are listed in Supplementary Table 1.

For RNase R treatment, 1 μg of total RNA was incubated for 15 min at 37 °C with or without 3 U of RNase R (Epicentre Technologies, Madison, WI). For actinomycin D treatment, cells were treated with 2 μg/ml actinomycin D or DMSO and collected after 24 h.

### Tartrate-resistant acid phosphatase (TRAP) staining

BMMs with the indicated treatment were seeded in a 96-well plate and cultured in the presence of M-CSF and RANKL for 5 days. Afterward, TRAP staining was performed using a TRAP staining kit (Sigma‒Aldrich, 387A) following the manufacturer’s protocol. TRAP-positive cells with two or more nuclei were considered osteoclasts and were counted.

### Bone resorption pit assay

BMMs with the indicated treatment were cultured in the presence of M-CSF and RANKL for 5 days to allow the formation of mature osteoclasts. Then, the cells were dissociated using cell dissociation buffer (Gibco, 13151014), reseeded on dentin slices in a 96-well plate, and cultured together with M-CSF and RANKL for another 2 days. Then, the dentin slice was collected, and pit lesion staining was performed. The brown area representing the bone resorption area was measured using ImageJ software (http://imagej.nih.gov/ij/).

### Antisense oligonucleotides, siRNAs and lentivirus

For knockdown of target circular RNAs (circFam190a, circAtrnl1 and circMkln1), locked nucleic acid enhanced antisense oligonucleotides (LNA-ASOs) were custom-designed and purchased from Qiagen (Hilden, Germany). Three different sequences were designed for each target circRNA, and a negative control sequence was included. The sequence information is listed in Supplementary Table 1. The knockdown efficiency was validated via RT‒qPCR.

For the knockdown of HSP90β and FUS, siRNA was designed and purchased from General Biol Company (Chuzhou, China). The sequence is shown in Supplementary Table 1.

For the overexpression of circFam190a, Fus and AKT1, pCDH (acting as a control), pCDH-Fus, pCDH-circFam190a, and pCDH-AKT1 plasmids were constructed and purchased from General Biol Company (Chuzhou, China). Lentiviral particles were produced by cotransfecting HEK-293T cells with these plasmids and helper plasmids (psPAX2 and pMD2.G) using Lipofectamine 3000 reagent (Thermo Fisher Scientific, Waltham, USA). The harvested supernatant was filtered through a 0.45-μm filter and used as a rich viral source for later cell transduction.

### ASO treatment in vitro and in vivo

For in vitro cell transfections, 2 × 10^5^ cells (BMMs or BMSCs) were seeded in a 6-well plate and transfected by using Lipofectamine 3000 (Thermo Fisher Scientific, Waltham, USA) following the manufacturer’s protocol. A mix of the indicated ASOs was used at a final concentration of 30 nM, and the cells were incubated for 24 h.

For in vivo animal experiments, eight-week-old female mice underwent either OVX or sham surgery. One week later, ASO-ctrl (negative control) or ASO-1 (targeting circFam190a) was injected into the lateral tail vein of the mice at a dose of 50 mg/kg twice a week as previously described^22^. Five weeks after the first injection, the mice were harvested. BMMs were collected for the validation of the knockdown efficiency. The left femur was sent for micro-CT analysis, the right femur was sent for bone histomorphometric analysis, and serum samples were collected for CTX1 and P1NP analysis.

### AAV and MK-2206 treatment

In vivo overexpression of circFam190a was achieved using AAV9 vectors. Murine circFam190a-overexpression AAV9 (AAV-circFam190a) and the corresponding control AAV9 (AAV-ctrl) were manufactured by GeneChem (Shanghai, China). Eight-week-old mice received a tail vein injection of 1 × 10^11^ viral particles of AAV-circFam190a or AAV-ctrl. After one week, the mice were intravenously treated with vehicle (PBS) or MK-2206 (120 mg/kg) three times per week for five weeks. The mice were then harvested, and BMMs, serum and femurs were collected for further analysis.

### Human sample collection

All protocols involving human samples were approved by the Research Ethics Committees of the First Affiliated Hospital of USTC (ethical approval no. 2000ky19). The procedures complied with the principles of the Declaration of Helsinki. Fifty postmenopausal women were randomly selected from the physical examination center at the First Affiliated Hospital of USTC. The inclusion criteria were postmenopausal women aged between 50 and 65 years with informed consent. Exclusion criteria included a history of alcohol abuse, severe renal or hepatic diseases, hyperthyroidism, chronic use of corticosteroids, severe gastrointestinal diseases, malignant tumors, diabetes mellitus, severe cardiac diseases, rheumatoid arthritis, other rheumatic-inflammatory diseases, and treatment for osteoporosis or osteopenia such as selective estrogen receptor modulators, bisphosphonate, and calcitonin.

In our study, all subjects were divided into three groups based on different BMD manifested by *T* score^23^. The “osteoporosis” group refers arbitrarily to those with *T* values below −2.5, the “osteopenia” group has values between −1.0 and −2.5, and the “normal” group has values above −1.0. Blood samples from each patient were taken in the morning (after an overnight fast) between 8 a.m. and noon using precooled tubes. Peripheral blood mononuclear cells (PBMCs) were isolated freshly by standard Ficoll (GE Healthcare, Little Chalfont, United Kingdom) density gradient centrifugation. Total RNA from PBMCs was isolated and stored at −80 °C for further analysis of circFAM190A expression. Detailed information about the patients is presented in Supplementary Table 2.

### Micro-CT analysis

For micro-CT analysis, a high-resolution μCT35 desktop microtomographic imaging system (Scanco Medical AG, Brüttisellen, Switzerland) was used. The left femurs from mice were collected for μCT scanning. Scans were acquired with a 12 µm^3^ isotropic voxel size, 55 kVP, 145 μA and a 600 ms integration time. The images were subjected to Gaussian filtration and segmented using a fixed threshold of 700 mgHA/cm^3^ as previously described^18^. The image acquisition and analysis protocols followed the JBMR guidelines^24^. The region of interest (ROI) was set as 100 μCT slices 1200 μm under the growth plate. The following variables were computed: bone mineral density (BMD), bone volume/total volume (BV/TV), trabecular bone number (Tb.N), trabecular bone thickness (Tb.Th), trabecular bone separation (Tb.Sp), and connectivity density (Conn.D). μCT images were reconstructed using built-in software.

### Bone histomorphometric analysis

For bone histomorphometric analysis, mice were injected with calcein and Alizarin red 4 days and 2 days prior to sacrifice, respectively. The right femurs were collected, fixed in 70% ethanol, and embedded as previously described^25,26^. Then, 5 μm standard undecalcified sections were cut using a microtome (Leica RM2255). von Kossa staining, toluidine blue staining and TRAP staining were performed. Quantitative bone histomorphometric measurements were performed using the OsteoMeasure system. The following variables were analyzed: bone volume/total volume (BV/TV), trabecular bone thickness (Tb.Th), trabecular bone space (Tb.Sp), trabecular bone number (Tb.N), osteoclast surface/bone surface (Oc.S/BS), osteoclast number/bone surface (Oc.N/BS), mineral apposition rate (MAR), and bone formation rate/bone surface (BFR/BS).

### CTX1 and P1NP assay

Mouse serum was collected after a 6-hour fasting period. The serum levels of the bone resorption marker collagen C-terminal telopeptide (CTX-1) and bone formation marker procollagen 1 N-terminal peptide (P1NP) were measured using ELISA kits (AC-06F1 and AC-33F1, Immunodiagnostics Systems, Maryland, USA) following the manufacturer’s instructions.

### Western blot (WB) and immunoprecipitation (IP)

For WB analysis, whole cell lysates were collected using RIPA buffer containing protease inhibitors (P1008, Beyotime, Shanghai, China). After boiling, the supernatants were subjected to SDS‒PAGE and transferred to membranes. The bands were visualized using enhanced chemiluminescence (E412–01, Vazyme, Nanjing, China). For IP analysis, cells were lysed in co-IP buffer with a protease inhibitor cocktail for 40 minutes on ice. Cell lysates were incubated with the indicated antibodies adsorbed to protein A/G Agarose (20421, Thermo Fisher Scientific, Waltham, USA) for 4 h at 4 °C, followed by three washes in co-IP buffer and elution at 95 °C for 10 min. The antibodies used are listed in Supplementary Table 3.

### RNA immunoprecipitation (RIP)

RIP experiments were performed using the Magna RIP RNA-Binding Protein Immunoprecipitation Kit (17-700, Millipore, Bedford, USA). Approximately 10^7^ cells were collected with RIP lysis buffer (approximately 100 μl) containing protease and RNase inhibitors. The cell lysates were then incubated with 5 μg of AGO2, the indicated antibodies, or control rabbit IgG (31235, Thermo Fisher Scientific, Waltham, USA)-coated beads with rotation at 4 °C overnight. After treatment with proteinase K, the immunoprecipitated RNAs were extracted using the RNeasy MinElute Cleanup Kit (Qiagen) and reverse transcribed using PrimeScript RT Master Mix (TaKaRa).

### Fluorescence in situ hybridization (FISH)

Cy3-labeled circFam190a probes (Supplementary Table 1) were synthesized by TSINGKE (Wuhan, China). The circFam190a FISH was performed as described previously^27^. Briefly, cells were fixed with the fixative solution, followed by permeabilization. Hybridization was performed at 37 °C overnight in a dark moist chamber. After being washed three times in 2×SSC (Solarbio, Beijing, China) for 10 min, the coverslips were sealed with parafilm containing DAPI. The images were acquired using a confocal laser scanning microscope (LSM 780, Carl Zeiss).

### RNA pulldown assays and spectrometry analysis

Biotin-labeled circFam190a (antisense) and control (sense) probes (Supplementary Table 1) were synthesized by TSINGKE (Wuhan, China). RNA pulldown assays were performed as previously described. Briefly, 10^7^ cells were washed in ice-cold phosphate-buffered saline, lysed in 500 μl of co-IP buffer, and incubated with 3 μg biotinylated DNA oligo probes at room temperature for 2 h. A total of 50 μl of washed streptavidin C1 magnetic beads (Invitrogen) was added to each binding reaction and further incubated at room temperature for another hour. The beads were washed briefly with co-IP buffer five times. The bound proteins in the pulldown materials were analyzed by mass spectrometry or Western blotting. Mass spectrometry analysis was performed by Oebiotech (Shanghai, China).

### Two-step RNA-binding protein immunoprecipitation

Two-step IP analysis was performed as previously described^28^. Briefly, cell lysates were collected using lysis buffer (20 mM HEPES (pH 7.8), 400 mM KCl, 5% glycerol, 5 mM EDTA, 1% NP40, protease inhibitor cocktail and RNase inhibitor). The lysates were first immunoprecipitated with anti-Flag antibodies before elution with Flag peptides. Ten percent of the sample was collected for WB and RT‒PCR analysis, and the rest of the eluate was further incubated with control IgG or anti-HA antibodies for second-phase immunoprecipitation.

### Luciferase reporter and mammalian two-hybrid assays

Mammalian two-hybrid assays were performed as previously described^28^. Assays were performed according to the manufacturer’s instructions (E2440, Promega, Madison, USA) after transfection of the indicated plasmids. For the mammalian two-hybrid assay, complementary DNAs for HSP90β and AKT1 were cloned into the pBIND and pACT vectors and transfected along with firefly (pG5luc) luciferase vectors. Reporter activities were measured 48 h later for the luciferase reporter and mammalian two-hybrid assays using the Dual-Luciferase Reporter Assay Kit (Promega). Renilla measurements were used to normalize changes in firefly luciferase activity.

### Sequencing data accession

The raw sequence data reported in this paper have been deposited in the Genome Sequence Archive (Genomics, Proteomics & Bioinformatics 2021) in the National Genomics Data Center (Nucleic Acids Res 2022), China National Center for Bioinformation/Beijing Institute of Genomics, Chinese Academy of Sciences (GSA: CRA009230) and are publicly accessible at https://ngdc.cncb.ac.cn/gsa^29,30^.

### Statistical analysis

Data are expressed as the mean ± SEM. Statistical analysis was conducted using unpaired two-tailed Student’s *t* test. For comparison of three or more groups, two-way ANOVA followed by Tukey’s multiple comparisons test for all groups was used. GraphPad Prism 9 was used for statistical analysis. A *P* value <0.05 was considered statistically significant.

## Results

### Identification of circFam190a, a circular RNA implicated in osteoclastogenesis

**Fig. 1:**
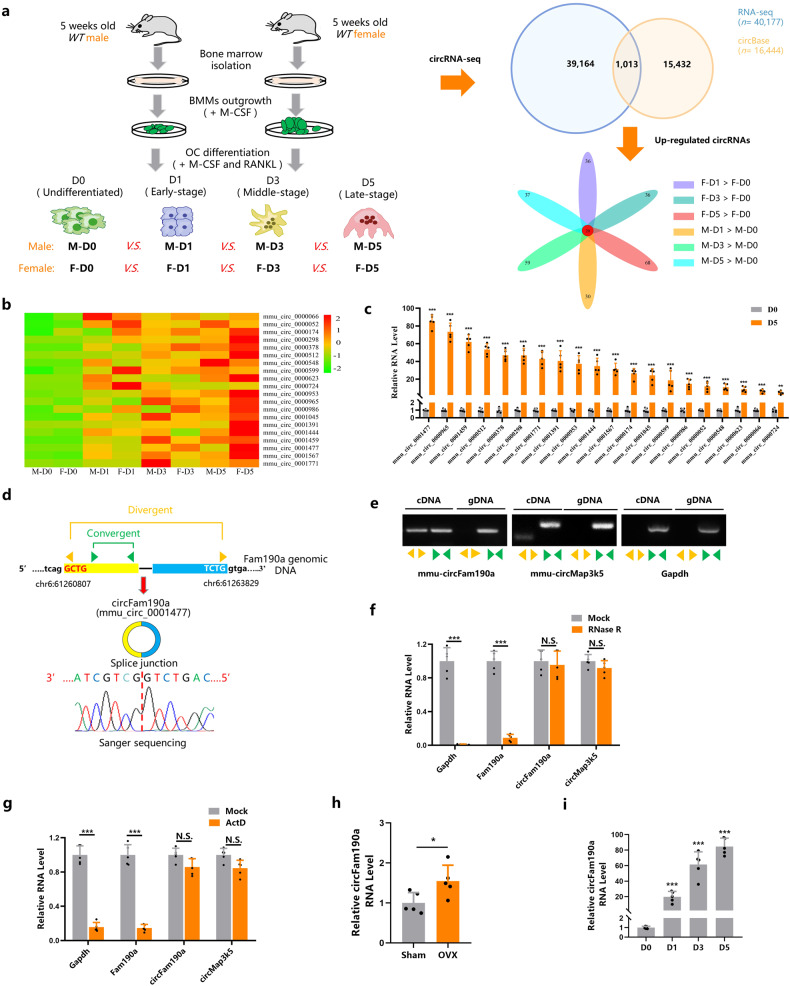
Identification of circFam190a, a circular RNA implicated in osteoclastogenesis. **a** Scheme of the strategies employed for circRNA-seq and circRNA screening. **b** Heatmap illustrating the continuously upregulated circRNAs. **c** qRT‒PCR analysis of selected circRNAs in undifferentiated (D0) and late-stage (D5) osteoclasts. Statistical significance: ***P < 0.001, **P < 0.01 (*n* = 5). **d** Sequencing analysis of the head-to-tail splicing junction in circFam190a. **e** Validation of the existence of circFam190a in osteoclasts using qRT‒PCR. Divergent primers amplified circFam190a in cDNA but not genomic DNA (gDNA). CircMap3k5 served as a positive control, and Gapdh was used as a negative control. Yellow arrows indicate divergent primers, while green arrows indicate convergent primers. Representative of three independent experiments. **f** qRT‒PCR analysis of relative RNA levels in osteoclasts treated with or without RNase R. Statistical significance: ***P < 0.001, N.S., not significant, *n* = 5. **g** qRT‒PCR analysis of relative RNA levels in osteoclasts treated with or without ActD. Statistical significance: ***P < 0.001, N.S., not significant, *n* = 5. **h** qRT‒PCR analysis of the relative circFam190a RNA levels in BMMs isolated from the sham and OVX mice. Statistical significance: *P < 0.05, (*n* = 5). **i** qRT‒PCR analysis of relative circFam190a RNA levels during osteoclast differentiation. Statistical significance: ***P < 0.001 compared to D0 (*n* = 5).

To investigate crucial circRNAs involved in osteoclastogenesis, we employed a sophisticated osteoclast in vitro culture system^31,32^. Samples were collected at different osteoclast differentiation stages: D0 (undifferentiated), D1 (early-stage OC differentiation), D3 (middle-stage OC differentiation), and D5 (late-stage OC differentiation) [Supplementary Fig. 1a]. Circular RNA sequencing analyses (circRNA-Seq) were performed to derive a list of expressed circRNAs [Fig. 1a, left]. Among a total of 40,178 identified circRNAs [Supplementary Fig. 1b], 1013 were annotated in circBase^33^ [Fig. 1a, upper right]. These annotated circRNAs were further screened, providing us with a list of 20 circRNAs referred to as “continuously upregulated circRNAs during osteoclastogenesis” [Fig. 1a, lower right, and Fig. 1b].

**Fig. 2:**
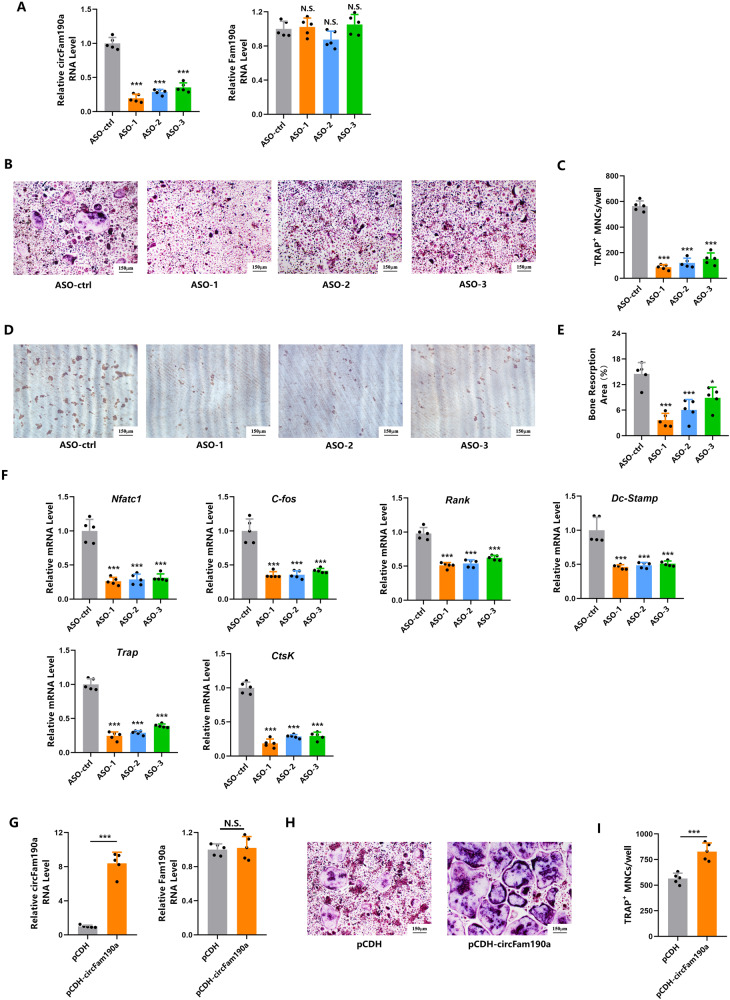
CircFam190a promotes osteoclast formation and function in vitro. **A** Expression levels of circRNA circFam190a and linear RNA Fam190a in BMMs transfected with ASOs. Statistical significance: ***P < 0.001 compared to the ASO-ctrl group, N.S., not significant, *n* = 5. **B**, **C** TRAP staining (**B**) and quantification (**C**) of osteoclasts in the control and circFam190a knockdown groups. Statistical significance: ***P < 0.001 compared to the ASO-ctrl group, *n* = 5. **D**, **E** Bone resorption pit assay: representative images of dentin slices seeded with control or circFam190a knockdown osteoclasts (**D**) and quantification (**E**) of the bone resorption area. The brown area indicates the bone resorption area. Statistical significance: ***P < 0.001 compared to the ASO-ctrl group, *P* < 0.05 compared to the ASO-ctrl group, *n* = 5. **F** Relative RNA levels of osteoclast-specific genes in the control and circFam190a knockdown osteoclasts. Statistical significance: ***P < 0.001 compared to the ASO-ctrl group, *n* = 5. **G** Expression levels of circRNA circFam190a and linear RNA Fam190a in BMMs transfected with pCDH (control) and pCDH-circFam190a. Statistical significance: ***P < 0.001 compared to the control group, N.S., not significant, *n* = 5. **H**, **I** TRAP staining (**H**) and quantification (**I**) of osteoclasts in the control and circFam190a overexpression groups. Statistical significance: ***P < 0.001 compared to the control group, N.S., not significant, *n* = 5.

We next validated the expression of these 20 circRNAs [Supplementary Fig. 1c]. Consistent with the sequencing results, qPCR data indicated significant upregulation of all 20 circRNAs in mature osteoclasts [Fig. 1c]. Among them, circFam190a (mmu_circ_0001477), circAtrnl1 (mmu_circ_0000965), and circMkln1 (mmu_circ_0001459) exhibited the most significant elevation. Notably, only the depletion of circFam190a, and not circAtrnl1 or circMkln1, ameliorated osteoclast formation [Supplementary Fig. 1d–f and Fig. 2A–C]. Hence, circFam190a captured our attention.

CircFam190a (mmu_circ_0001477) is generated from the *Fam90a* gene located on mouse chromosome (chr6). The annotation for circFam190a depicts 2 and 3 exons of *Fam190a* (total 1541 bp)^33^. We sought to verify that the form of circFam190a expressed by osteoblasts matches this report. First, sequencing confirmed that the head-to-tail splice junction was identical to the reported sequence [Fig. 1d]. Subsequently, the head-to-tail splicing of endogenous circFam190a was assessed using RT‒PCR with convergent and divergent primers, along with circMap3k5 as a control^34^. Consistent with the circular form, the divergent primers for circFam190a and circMap3k5, but not Gapdh, amplified a PCR product [Fig. 1e]. Furthermore, qPCR analysis of total RNA after RNase R treatment indicated that circFam190a and circMap3k5 were resistant, while Gapdh and Fam190a mRNA transcripts were degraded [Fig. 1f]. Treatment with actinomycin D suggested that circFam190a transcripts were more stable than Fam190a mRNA [Fig. 1g]. These findings established that circFam190a is a bona fide circRNA.

Notably, the sequence of circFam190a in mice (mmu_circ_0001477) exhibits high homology with its human counterpart (hsa_circ_0127310) [Supplementary Fig. 1g]. Similar to mouse circFam190a, hsa_circ_0127310 (referred to as circFAM190A) is generated from the human *FAM190A* gene, located on human chr 4^33^ [Supplementary Fig. 1h]. We also validated the expression of circFAM190A [Supplementary Fig. 1i–k]. Interestingly, in mice with ovariectomy surgery, a condition characterized by hyperactive osteoclasts, circFam190a expression was significantly elevated [Fig. 1h]. Consistent with these results, the expression level of circFAM190A in postmenopausal osteoporosis (PMO) patients was also significantly upregulated [Supplementary Fig. 1l]. Moreover, in vitro culture studies demonstrated a significant increase in circFam190a expression during Rankl-induced osteoclastogenesis [Fig. 1i].

Taken together, these in vivo and in vitro findings establish circFam190a as a bona fide circRNA that is upregulated during osteoclastogenesis, implying a potential correlation between elevated circFam190a/circFAM190A levels and excessive osteoclast activity.

### CircFam190a promotes osteoclast formation and function in vitro

As shown, the knockdown of circFam190a resulted in a significant drop in the number of osteoclasts formed [Fig. 2A–C]. Moreover, the depletion of circFam190a impaired osteoclast bone resorption function, as evidenced by the reduction in bone resorption area [Fig. 2D, E]. Consistently, gene expression analysis revealed a significant decrease in the expression of osteoclast-specific genes, including Nfatc1, Ctsk, C-fos, Rank, Trap, and Ctsk, upon blocking circFam190a [Fig. 2F]. These results indicate that the depletion of circFam190a inhibits osteoclast differentiation and function.

Conversely, the overexpression of circFam190a enhanced osteoclast formation and function, as demonstrated by an increased osteoclast formation number, an expanded bone resorption area, and upregulation of osteoclast-related genes [Fig. 2G–I and Supplementary Fig. 2].

Collectively, these data demonstrate that circFam190 acts as a positive regulator of osteoclast formation and function in vitro.

### Knockdown of circFam190a protects mice from pathological bone loss in an osteoporosis mouse model

**Fig. 3:**
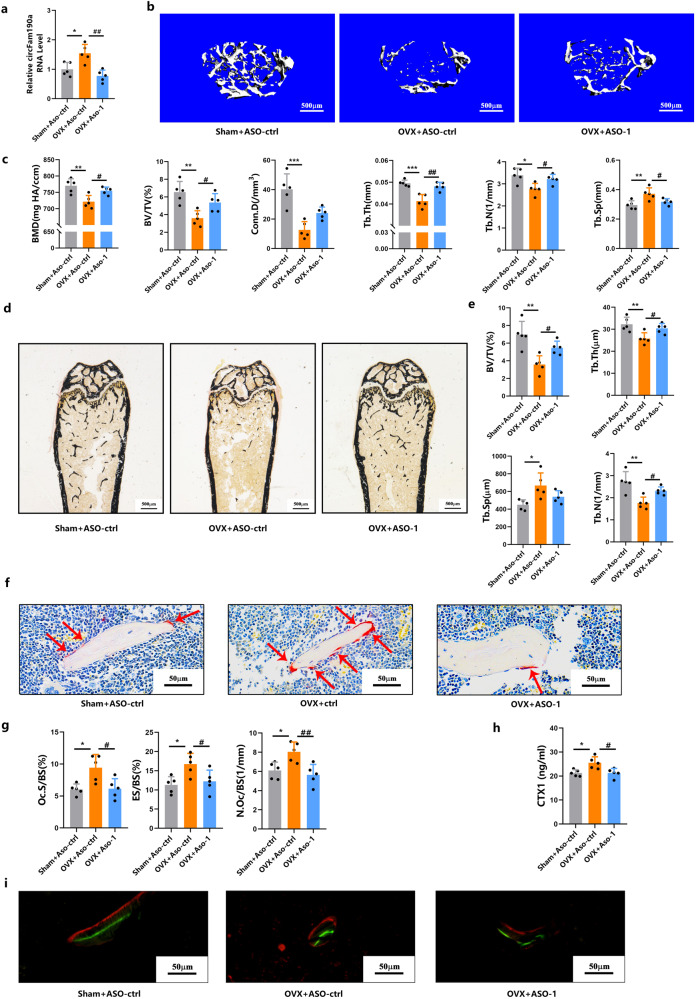
Knockdown of circFam190a protects mice from pathological bone loss in an osteoporosis mouse model. **a** Relative circFam190a RNA levels in BMMs collected from the indicated mice. Statistical significance: *P < 0.05 compared to the sham+ASO-ctrl group, ^##^*P* < 0.01 compared to the OVX + ASO-ctrl group, *n* = 5. **b** Representative micro-CT images of mouse left femurs. **c** Quantification of bone parameters using micro-CT. BMD bone mineral density, BV/TV bone volume/total volume, Tb.N trabecular bone number, Tb.Th trabecular bone thickness, Tb.Sp trabecular bone separation, Conn.D connectivity density. Statistical significance: ***P < 0.001 compared to the sham+ASO-ctrl group, **P < 0.01 compared to the sham+ASO-ctrl group, *P < 0.05 compared to the sham+ASO-ctrl group, ^##^*P* < 0.01 compared to the OVX + ASO-ctrl group, ^#^*P* < 0.05 compared to the OVX + ASO-ctrl group, *n* = 5. **d** Representative von Kossa staining images of undecalcified sections from the right femurs of mice. **e** Histomorphometric analysis of undecalcified sections from the right femurs of mice. BV/TV bone volume/total volume, Tb.Th trabecular bone thickness, Tb.Sp trabecular bone space, Tb.N trabecular bone number. Statistical significance: **P < 0.01 compared to the sham+ASO-ctrl group, *P < 0.05 compared to the sham+ASO-ctrl group, ^#^*P* < 0.05 compared to the OVX + ASO-ctrl group, *n* = 5. **f** Representative TRAP staining images of undecalcified sections from the right femurs of mice. Red arrows indicate osteoclasts. **g** Histomorphometric analysis of osteoclast-related parameters: Oc. S/BS osteoclast surface/bone surface, ES/BS eroded surface/bone surface, Oc.N/BS osteoclast number/bone surface, Statistical significance: *P < 0.05 compared to the sham+ASO-ctrl group, ^##^*P* < 0.01 compared to the OVX + ASO-ctrl group, ^#^*P* < 0.05 compared to the OVX + ASO-ctrl group, *n* = 5. **h** Serum bone resorption marker CTX-1 levels in the indicated mice. Statistical significance: *P < 0.05 compared to the sham+ASO-ctrl group, ^#^*P* < 0.05 compared to the OVX + ASO-ctrl group, *n* = 5. **i** Representative double labeling images of trabecular bone from the right femurs of mice. The green line indicates the first injection of calcein, and the red line indicates the second injection of Alizarin red.

To further validate the role of circFam190a in osteoclasts, we expanded our experiments to include in vivo animal studies. Successful construction of the OVX mouse model, an estrogen deficiency-induced bone loss model, was confirmed by the decreased uterus weight [Supplementary Fig. 3a], and the efficiency of circFam190a knockdown in mice was validated by qRT‒PCR [Fig. 3a]^35,36^.

Microcomputed tomography (Micro-CT) analysis revealed that the depletion of circFam190a in mice ameliorated OVX-induced bone loss, as supported by increased BMD, BV/TV, Tb.Th, Tb.N, Conn.D, and decreased Tb.Sp levels [Fig. 3b, c]. Consistently, bone histomorphometric analysis also showed increased BV/TV, Tb.Th, and Tb.N and decreased Tb.Sp in mice with circFam190a depletion [Fig. 3d, e]. Moreover, histomorphometric results demonstrated that the knockdown of circFam190a prevented the increase in osteoclast number, osteoclast surface and erosion surface induced by OVX, indicating the inhibition of osteoclast activity [Fig. 3f, g]. This inhibitory effect was confirmed by the decreased serum CTX1 levels in mice with circFam190a deletion [Fig. 3h].

Interestingly, the knockdown of circFam190a did not affect bone formation, as evidenced by no significant differences in bone formation parameters (Ob.S/BS, N.Ob/BS, MAR and BFR/BS) and serum P1NP levels between the groups with or without circFam190a deletion [Fig. 3i and Supplementary Fig. 3b–d]. To further confirm this effect on bone formation, we also performed circFam190a knockdown during osteoblast differentiation in vitro [Supplementary Fig. 3e–h]. As shown, no differences in staining or expression of bone formation-related genes were observed when circFam190a was inhibited, indicating no effect of circFam190a on bone formation.

Collectively, these results demonstrate that in vivo knockdown of circFam190a inhibits hyperactive osteoclasts induced by OVX without affecting bone formation.

### FUS increases circFam190a production during osteoclastogenesis

**Fig. 4:**
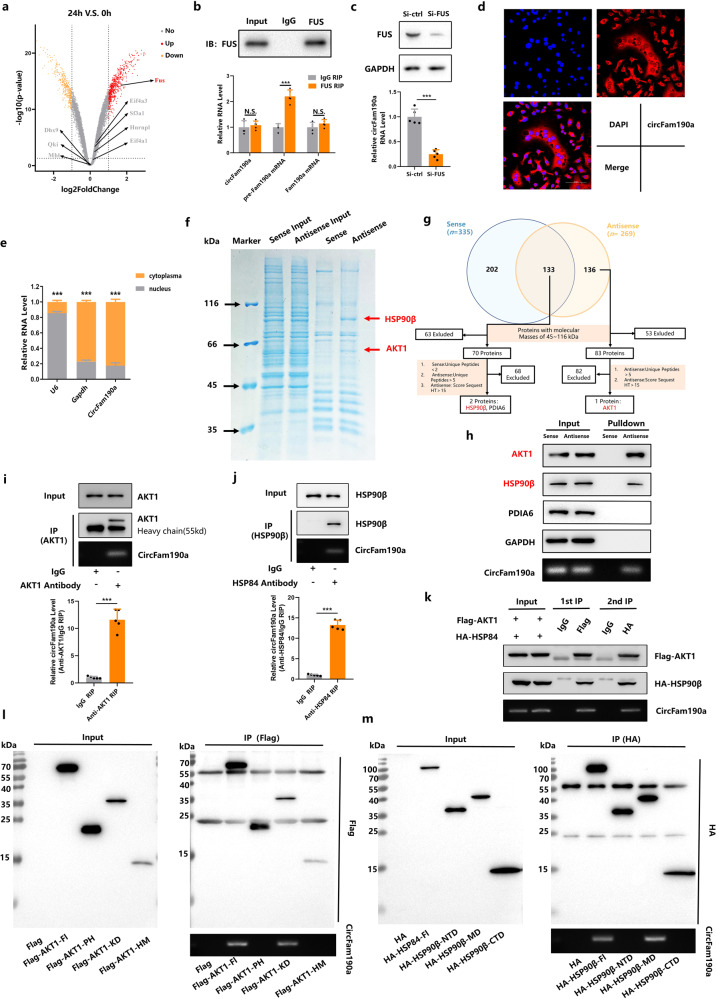
CircFam190a is regulated by FUS and binds directly to HSP90β/AKT1. **a** Volcano plot showing differential gene expression in BMMs treated with RANKL for 24 h vs. untreated BMMs (controls). Each scattered point represents a gene: the *x*-axis represents the log2-fold change in the ratio of RANKL-treated cells vs. untreated cells, and the y-axis represents the −log10 of the P value. Red dots indicate significantly upregulated genes, while yellow dots indicate significantly downregulated genes. **b** RIP assays of BMMs using FUS and IgG antibodies. The precipitate was subjected to WB analysis with FUS antibodies, and the relative RNA level was calculated by qRT‒PCR. Statistical significance: ***P < 0.001, N.S., not significant, *n* = 5. **c** Relative circFam190a RNA levels in osteoclasts with or without FUS knockdown. Statistical significance: ***P < 0.001, *n* = 5. **d** RNA-FISH staining assay of osteoclasts indicating the localization of circFam190a (red) with nuclei stained with DAPI (blue). The data shown are representative of three independent experiments. **e** Identification of circFam190a cytoplasmic and nuclear distribution by qRT‒PCR analysis in osteoclasts. GAPDH and U6 were applied as positive controls in the cytoplasm and nucleus, respectively. Statistical significance: ***P < 0.001, *n* = 5. **f** Biotin-labeled sense or antisense circFam190a probes were used for RNA‒protein pulldown against osteoclast lysates. Proteins that interact with circFam190a were identified by Coomassie brilliant blue staining. The red arrow indicates the major differential band precipitated. **g** Analysis pipeline used to identify proteins that interact with circFam190a. **h** Immunoblot analysis of the biotin-labeled sense and antisense circFam190a probe pulldown eluate from osteoclast lysates. GAPDH was used as a loading control. The data shown are representative of three independent experiments. **i** RIP assays of osteoclasts using AKT1 and IgG antibodies. The precipitate was subjected to WB with AKT1 antibodies. The relative RNA level was calculated by qRT‒PCR. Statistical significance: ***P < 0.001, *n* = 5. **j** RIP assays of osteoclasts using HSP90β and IgG antibodies. The precipitate was subjected to WB with HSP90β antibodies. The relative RNA level was calculated by qRT‒PCR. Statistical significance: ***P < 0.001, *n* = 5. **k** Two-step RNA-binding protein immunoprecipitation: osteoclasts cotransfected with Flag-AKT1 and HA-HSP90β were used to perform sequential immunoprecipitation with anti-Flag and anti-HA antibodies. The precipitate was subjected to WB with antibodies against Flag-AKT1 and HA-HSP90β. The data shown are representative of three independent experiments. **l** RIP assays of osteoclasts transfected with full-length AKT1 or its deletion mutants using Flag antibody. The circFam190a level was detected by semiquantitative RT‒PCR agarose gels. The data shown are representative of three independent experiments. **m** RIP assays of osteoclasts transfected with full-length HSP90β or its deletion mutants using HA antibody. The circFam190a level was shown by semiquantitative RT‒PCR agarose gel. The data shown are representative of three independent experiments.

Based on the aforementioned results confirming the importance of circFam190a as a positive regulator of osteoclastogenesis, we aimed to identify the upstream regulatory factors responsible for circFam190a production. Recently, several RNA-binding proteins (RBPs) have been reported to regulate circRNA biogenesis^37,38^. Considering the significant upregulation of circFam190a during osteoclast differentiation, we hypothesized that RBPs involved in circFam190a production would also increase following RANKL stimulation. Therefore, we screened the expression of 8 candidate circRNA biogenesis-associated RBPs (EIF4A1, EIF4A3, SF3A1, HNRNPL, FUS, DHX9, QKI and MBL) using transcriptome analysis of BMMs treated with M-CSF and RANKL compared to untreated BMMs (GSE176265)^39^. As shown in the volcano plot, only FUS showed a significant increase during osteoclastogenesis [Fig. 4a]. This upregulation of FUS during osteoclast differentiation was further confirmed by RT‒PCR data [Supplementary Fig. 4a]. Importantly, RNA immunoprecipitation (RIP) assays indicated that FUS could bind to pre-Fam190a mRNA but not circFam190a or Fam190a mRNA [Fig. 4b]. Moreover, knockdown of FUS resulted in a decrease in circFam190a levels, while ectopic expression of FUS increased circFam190a levels [Fig. 4c and Supplementary Fig. 4b]. These findings collectively indicate that the expression of circFam190a is driven by FUS.

### CircFam190a binds directly to HSP90β and AKT1

We then studied how circFam190a exerts its effect on osteoclasts. Since circFam190a was predominantly cytoplasmic [Fig. 4d, e], we first examined whether circFam190a functions as a miRNA sponge. Argonaut 2 crosslinking and immunoprecipitation (AGO2 CLIP) was performed^40^ with an AGO2 RIP assay. However, the results showed that circFam190a did not bind to AGO2 [Supplementary Fig. 4c], ruling out the possibility that circFam190a functions as a miRNA sponge.

To identify potential protein binding partners of circFam190a, we conducted RNA pulldown assays using biotinylated probes targeting the backspliced sequence of circFam190a [Fig. 4f]. Mass spectrometry analysis of the sense and antisense probes was performed following the analysis pipeline [Fig. 4g], identifying 3 candidate RNA binding proteins (RBPs). Subsequently, immunoblot analysis of the pulldown eluate confirmed the presence of AKT1 and HSP90β in the antisense eluate, while they did not bind to the sense probe [Fig. 4h]. Interestingly, previous reports have suggested that HSP90β can form a complex and interact with AKT1^41–45^. Our RIP analysis further confirmed a strong interaction between circFam190a and AKT1 as well as HSP90β [Supplementary Fig. 4d, e]. Consistently, RIP assays targeting endogenous AKT1 and HSP90β reaffirmed their interaction with circFam190a [Fig. 4i, j].

Seeking to further verify the nature of the association between circFam190a, AKT1 and HSP90β, we performed a two-step IP after transfecting FLAG-AKT1 and HA-HSP90β into osteoclasts. In the first phase, anti-FLAG antibodies precipitated FLAG-AKT1 along with HA-HSP90β and circFam190a from total protein extracts. Moreover, the secondary IP against HA-HSP90β captured FLAG-AKT1 and CircFam190a [Fig. 4k]. These results indicate the formation of a ternary complex involving circFam190a, AKT1, and HSP90β.

To further delineate the structural determinants of the interactions between circFam190a, AKT1 and HSP90β, we performed deletion mapping by subdividing the functional domains of AKT1 and HSP90β [Supplementary Fig. 4f, g]^46,47^. RNA pulldown assays showed that removal of the kinase domain (KD) (aa150-408) of AKT1 abolished its association with circFam190a [Fig. 4l], while deletion of the middle domain (MD) (aa262-609) of HSP90β diminished its interaction with circFam190a [Fig. 4m].

In summary, these results indicate that circFam190a, AKT1 and HSP90β form an RNA‒protein trimer complex through the KD domain of AKT1 and the MD domain of HSP90β.

### CircFam190a protects AKT1 from proteasome-mediated degradation

**Fig. 5:**
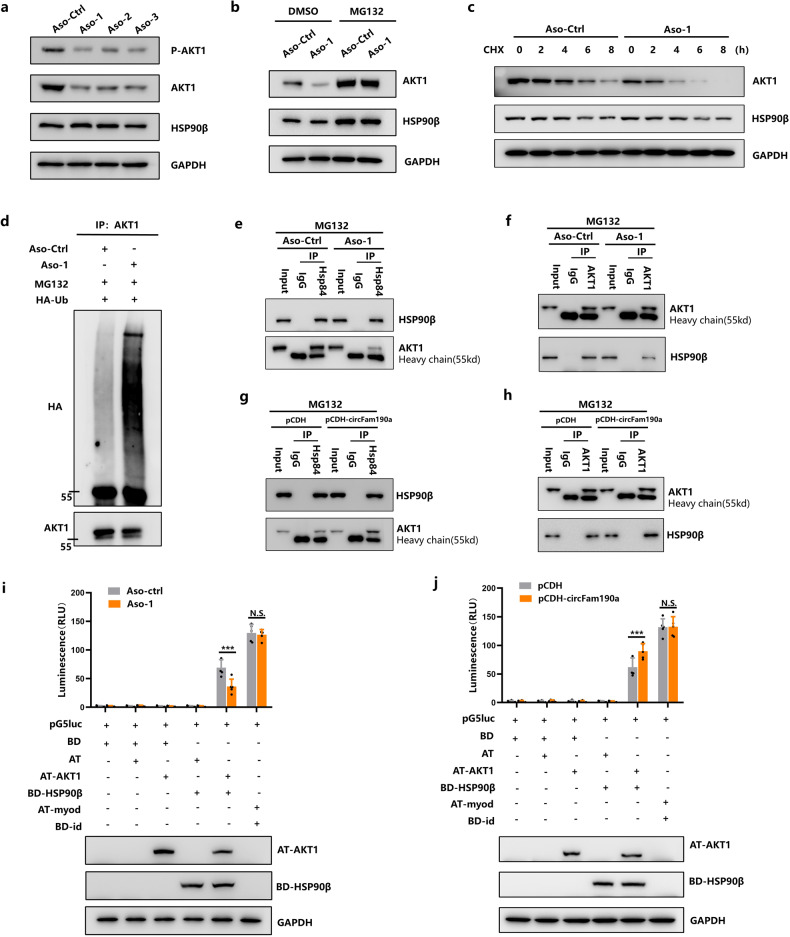
CircFam190a Protects AKT1 from Proteasome-Mediated Degradation. **a** BMMs transfected with control (ASO-ctrl) or circFam190a knockdown (ASO-1, ASO-2, ASO-3) LNA-ASOs were cultured with M-CSF and RANKL for 3 days. Whole cell lysates were collected for WB analysis. The data shown are representative of three independent experiments. **b** BMMs transfected with control ASO-ctrl or circFam190a knockdown (ASO-1) LNA-ASOs were treated with DMSO or 10 μM MG132 for 12 h. Whole cell lysates were collected for WB. The data shown are representative of three independent experiments. **c** Cycloheximide chase assay comparing the stability of AKT1 and HSP90β in BMMs transfected with ASO-ctrl or ASO-1 LNA-ASOs. The indicated cells were treated with cycloheximide (50 μg/ml) for the indicated times, and cell lysates were subjected to WB analysis with AKT1 and HSP90β antibodies, along with a GAPDH control. The data shown are representative of three independent experiments. **d** Ubiquitination assays of AKT1. BMMs transfected with control (ASO-ctrl) or circFam190a knockdown (ASO-1) LNA-ASOs were transfected with HA-Ub and treated with 10 μM MG132 for 8 h. The whole cell lysate was immunoprecipitated by AKT1 antibody. The precipitate was subjected to WB with antibodies against HA and AKT1. The data shown are representative of three independent experiments. **e**, **f** Immunoprecipitation analysis of the association between HSP90β and AKT1 in MG132-treated BMMs with the control (ASO-ctrl) or circFam190a knockdown (ASO-1) LNA-ASOs. The whole cell lysate was immunoprecipitated with IgG and HSP90β (**e**) or AKT1 (**f**) antibodies. The precipitate was subjected to WB with antibodies against HSP90β and AKT1. The data shown are representative of three independent experiments. **g**, **h** Immunoprecipitation analysis of the association between HSP90β and AKT1 in MG132-treated BMMs transfected with control (pCDH) or circFam190a overexpression (pCDH-circFam190a) plasmids. The whole cell lysate was immunoprecipitated with IgG and HSP90β (**g**) or AKT1 (**h**) antibodies. The precipitate was subjected to WB with antibodies against HSP90β and AKT1. The data shown are representative of three independent experiments. **i** Mammalian two-hybrid assays measuring interactions between AKT1 and HSP90β with or without circFam190a knockdown. The indicated combinations of pBIND/pACT plasmids were transfected into BMMs with either the control ASO (ASO-ctrl) or circFam190a knockdown ASO (ASO-1) before measuring relative luciferase activity (a proxy for the interaction between proteins). The presence of transfected fusion proteins was verified by Western blot analysis of cell lysates. Statistical significance: ***P < 0.001, N.S., not significant, *n* = 5. **j** Mammalian two-hybrid assays measuring interactions between AKT1 and HSP90β with or without overexpression of circFam190a. The indicated combinations of pBIND/pACT plasmids were transfected into BMMs bearing either a control plasmid (pCDH) or the circFam190a overexpression plasmid pCDH-circFam190a before measuring relative luciferase activity (a proxy for the interaction between proteins). The presence of the transfected fusion proteins was verified by Western blot analysis of cell lysates. Statistical significance: ***P < 0.001, N.S., not significant, *n* = 5.

Next, we sought to determine the effects of circFam190a on AKT1 and HSP90β. Knockdown of circFam190a led to a significant reduction in AKT1 protein levels without affecting HSP90β [Fig. 5a]. Conversely, overexpression of circFam190a upregulated AKT1 protein expression [Supplementary Fig. 5a]. Additionally, treatment with the proteasome inhibitor MG132 stabilized AKT1 levels following circFam190a silencing or overexpression, indicating that circFam190a influences AKT1 protein stability [Fig. 5b and Supplementary Fig. 5a]. Consistently, circFam190a knockdown decreased the half-life of AKT1, as observed in cycloheximide chase assays, while HSP90β levels remained unaffected [Fig. 5c]. Additionally, circFam190a knockdown increased the polyubiquitination of AKT1 [Fig. 5d], suggesting that circFam190a prevents the ubiquitination and proteasomal degradation of AKT1 without affecting HSP90β.

It has been reported that HSP90β can form a complex with AKT1 and protect AKT1 from proteasome-mediated degradation^48^. Considering the formation of the circFam190a/HSP90β/AKT1 complex, we asked whether circFam190a could modulate the interactions between HSP90β and AKT1, thereby influencing AKT1 stability. As shown, silencing circFam190a significantly reduced the binding between HSP90β and AKT1 [Fig. 5e, f], while overexpression of circFam190a enhanced their binding [Fig. 5g, h]. As a complementary approach, we employed the mammalian two-hybrid system to further study the influence of circFam190 on AKT1-HSP90β protein‒protein interactions in vivo. Bait (BD) and prey (AT) fusion vectors of AKT1 and HSP90β were constructed, and the interactions were measured by reporter assays. The results showed that circFam190a silencing reduced the interaction between HSP90β and AKT1 [Fig. 5i], while circFam190a overexpression enhanced their binding [Fig. 5j].

Collectively, these findings indicate that circFam190a promotes the binding of AKT1 and HSP90β, thereby facilitating AKT1 stability and activity.

### CircFam190a promotes osteoclast formation in an AKT1-dependent manner in vitro

**Fig. 6:**
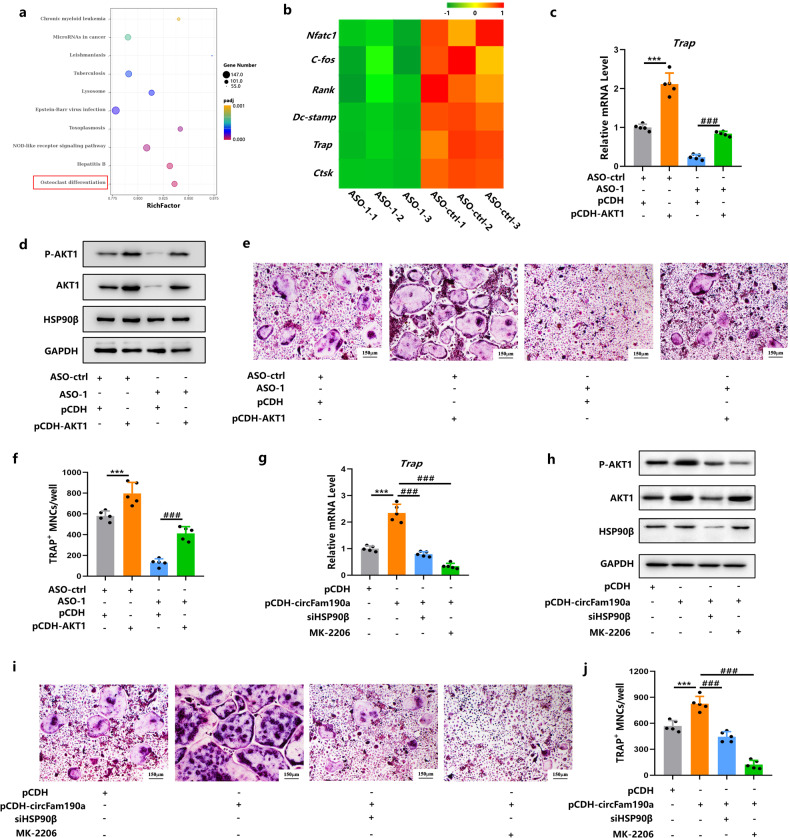
CircFam190a promotes osteoclast formation in an AKT1-dependent manner in vitro. **a**, **b** KEGG pathway enrichment analysis (**a**) and heatmap of osteoclast-related genes (**b**) in transcriptome analysis comparing osteoclasts with or without circFam190a knockdown. BMMs with the control (ASO-ctrl) or circFam190a knockdown (ASO-1) LNA-ASOs were cultured with M-CSF and RANKL for 72 h to induce osteoclast differentiation. Whole RNA samples were collected and subjected to transcriptome analysis. **c**, **d** Relative TRAP RNA levels (**c**) and WB analysis (**d**) of osteoclasts with or without circFam190a knockdown and AKT1 overexpression. BMMs were transfected with the indicated ASOs (ASO-ctrl or ASO-1) and plasmids (pCDH or pCDH-AKT1) and cultured with M-CSF and RANKL for 72 hours to induce osteoclast differentiation. For mRNA analysis, ***P < 0.001 compared to the ASO-ctrl + pCDH group, ^###^*P* < 0.001 compared to the ASO-1 + pCDH group, *n* = 5. For WB, the data shown are representative of three independent experiments. **e**, **f** TRAP staining (**e**) and quantification (**f**) of osteoclasts with or without circFam190a knockdown and AKT1 overexpression. BMMs were transfected with the indicated ASOs (ASO-ctrl or ASO-1) and plasmids (pCDH or pCDH-AKT1) and cultured with M-CSF and RANKL for 5 days to induce mature osteoclast formation. Statistical significance: ***P < 0.001 compared to the ASO-ctrl + pCDH group, ^###^*P* < 0.001 compared to the ASO-1 + pCDH group, *n* = 5. **g**, **h** Relative TRAP RNA levels (**g**) and WB analysis (**h**) of osteoclasts with or without circFam190a overexpression and the indicated treatment. BMMs received the indicated treatment (pCDH, pCDH-circFam190a or/and siHSP90β) and were cultured with or without MK-2206 in the presence of M-CSF and RANKL for 72 h. For mRNA analysis, ***P < 0.001 compared to the pCDH group, ^###^*P* < 0.001 compared to the pCDH-circFam190a group, n = 5. For WB, the data shown are representative of three independent experiments. **i**, **j** TRAP staining (**i**) and quantification (**j**) of osteoclasts with or without circFam190a overexpression and the indicated treatment. BMMs received the indicated treatment (pCDH, pCDH-circFam190a or/and siHSP90β) and were cultured with or without MK-2206 in the presence of M-CSF and RANKL for 5 days. Statistical significance: ***P < 0.001 compared to the pCDH group, ^###^*P* < 0.001 compared to the pCDH-circFam190a group, *n* = 5.

After confirming the effects of circFam190a on AKT1, we next asked whether circFam190a exerted its function on osteoclasts in an AKT1-dependent manner. mRNA sequencing analyses were performed on control and circFam190a-silenced osteoclast samples [Supplementary Fig. 6a, b]. Consistent with its role as a positive regulator of osteoclast differentiation, circFam190a knockdown led to a significant decrease in the expression of osteoclast differentiation-related genes [Fig. 6a, b].

Moreover, the overexpression of AKT1 successfully rescued the decreased osteoclast formation induced by circFam190a silencing [Fig. 6c–f and Supplementary Fig. 6c]. To further confirm the involvement of AKT1 signaling, we conducted additional experiments. Treatment with MK-2206, an inhibitor of the AKT1 signaling pathway, or depletion of HSP90β using siHSP90β, which is involved in stabilizing AKT1, both rescued the hyperactive osteoclast phenotype induced by circFam190a overexpression [Fig. 6g–j and Supplementary Fig. 6d].

These findings collectively suggest that circFam190a promotes osteoclast formation in an AKT1-dependent manner in vitro. The upregulation of circFam190a enhances osteoclastogenesis through the activation of AKT1 signaling, and the inhibition of AKT1 signaling or disruption of the circFam190a-AKT1 interaction attenuates osteoclast formation.

### Inhibition of AKT signaling rescues the osteoclast phenotype induced by circFam190a in vivo

**Fig. 7:**
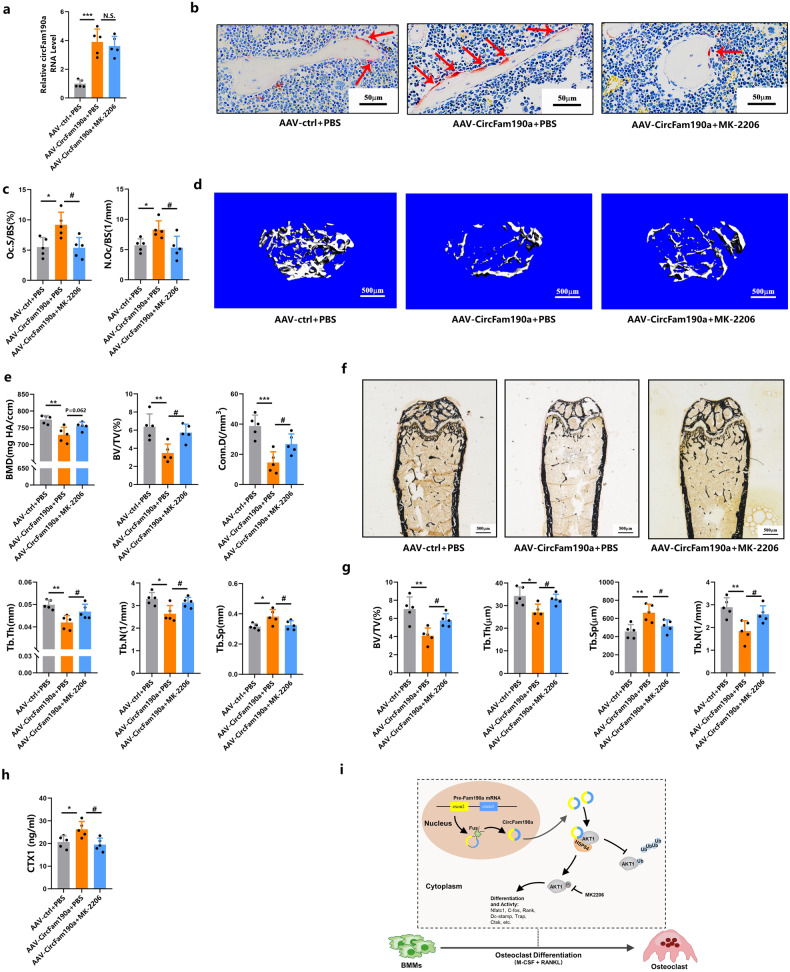
Inhibition of AKT signaling rescues the osteoclast phenotype induced by circFam190a in vivo. **a** Relative circFam190a RNA levels in BMMs collected from the indicated mice. Statistical significance: ***P < 0.001 compared to the AAV + PBS group, N.S., not significant, *n* = 5. **b** Representative TRAP staining images of undecalcified sections from the right femurs of mice. Red arrows indicate osteoclasts. **c** Histomorphometric analysis of osteoclast-related parameters: Oc.S/BS: osteoclast surface/bone surface, Oc.N/BS: osteoclast number/bone surface. Statistical significance: *P < 0.05 compared to the AAV + PBS group, ^#^*P* < 0.05 compared to the AAV-CircFam190a+PBS group, N.S., not significant, *n* = 5. **d** Representative micro-CT images of mouse left femurs. **e** Quantification of bone parameters by micro-CT. BMD bone mineral density, BV/TV bone volume/total volume, Tb.N trabecular bone number, Tb.Th trabecular bone thickness, Tb.Sp trabecular bone separation, Conn.D connectivity density. Statistical significance: *P < 0.05 compared to the AAV + PBS group, **P < 0.01 compared to the AAV + PBS group, ***P < 0.001 compared to the AAV + PBS group, ^#^*P* < 0.05 compared to the AAV-circFam190a+PBS group, N.S., not significant, *n* = 5. **f** Representative von Kossa staining images of undecalcified sections from the right femurs of mice. **g** Histomorphometric analysis of undecalcified sections from the right femurs of mice. BV/TV bone volume/total volume, Tb.Th trabecular bone thickness, Tb.Sp trabecular bone space, Tb.N trabecular bone number, statistical significance: *P < 0.05 compared to the AAV + PBS group, **P < 0.01 compared to the AAV + PBS group, ^#^*P* < 0.05 compared to the AAV-circFam190a+PBS group, *n* = 5. **h** Serum bone resorption marker CTX-1 levels from the indicated mice. Statistical significance: *P < 0.05 compared to the AAV + PBS group, ^#^*P* < 0.05 compared to the AAV-circFam190a+PBS group, *n* = 5. **i** Working model of circFam190a as a critical positive regulator during osteoclastogenesis.

To further validate the role of AKT signaling in mediating the effects of circFam190a on osteoclasts in vivo, we used adeno-associated virus (AAV) to overexpress circFam190a in mice. Successful overexpression of circFam190a was validated in trabecular bone samples [Fig. 7a]. Since AAV9, when intravenously injected, can also transduce other tissues such as the liver, heart, and muscle^49,50^, the expression of circFam190a in these tissues was also examined [Supplementary Fig. 7a].

Our findings in Fig. 3 showed that the depletion of circFam190a in vivo inhibited osteoclast bone resorption [Fig. 3]. Consistent with this finding, the overexpression of circFam190a in vivo induced hyperactive osteoclasts, as indicated by the significantly elevated OC number and surface [Fig. 7b, c], which led to a significant decline in bone volume [Fig. 7d–g]. Importantly, treatment with MK-2206, an inhibitor of AKT signaling, successfully rescued the bone loss induced by circFam190a overexpression [Fig. 7d–g]. This protective effect was attributed to the inhibition of osteoclast function, as evidenced by the significant decrease in osteoclast number and surface area following MK-2206 treatment [Fig. 7b, c]. Consistently, serum CTX1 levels were also decreased after MK-2206 treatment [Fig. 7h].

Bone formation parameters were also evaluated in these mice. The data showed no significant difference with or without circFam190a overexpression, indicating that circFam190a did not affect bone formation [Supplementary Fig. 7a–d]. However, treatment with MK-2206 impaired bone formation, as indicated by the significant decrease in parameters such as Ob.S/BS, N.Ob/BS, MAR, BFR/BS, and serum P1NP levels [Supplementary Fig. 7a–d]. This inhibitory effect of MK-2206 on bone formation is reasonable considering that MK-2206 can also impair the AKT signaling pathway in osteoblasts.

In summary, these results suggest that the overexpression of circFam190a induces hyperactive osteoclast-mediated bone resorption. This phenotype can be rescued by inhibiting AKT signaling with MK-2206, thereby protecting mice from circFam190a-induced bone loss.

## Discussion

Although the physiological roles of osteoclasts are well described, the mechanisms of their differentiation and function remain to be elucidated. In the current study, we identified circFam190a as a critical positive regulator of osteoclastogenesis [Fig. 7i]. Specifically, we found that circFam190a, induced by FUS, binds directly to AKT1 and HSP90β, facilitating their association. This interaction protects AKT1 from proteasomal degradation, thereby activating AKT1 signaling and promoting osteoclast formation and function [Fig. 7i].

CircRNAs exert their functions through several well-established mechanisms, including the regulation of parental gene expression and splicing events, regulation of gene expression through miRNA sponging, translation into proteins, and interaction with proteins^51,52^. Previous studies have reported that circ_0008542 promotes osteoclast differentiation and bone resorption by acting as a miR-185-5p sponge^53^, and circCHEK1_246aa stimulates osteoclast differentiation by encoding the CHEK1 kinase catalytic center^54^. However, it remains unclear whether circRNAs can regulate osteoclasts via circRNA-protein interactions. Our findings demonstrate that circFam190a can interact with HSP90β and AKT1, representing the first report of circRNAs functioning through circRNA-protein interactions in the regulation of osteoclastogenesis. This finding expands our understanding of the molecular mechanisms underlying circRNAs and osteoclastogenesis.

To unveil the role of circRNAs during osteoclastogenesis, we performed deep circRNA sequencing to identify the expression profiles of circRNAs at different stages of osteoclast differentiation (D0, before RANKL stimulation; D1, 1 day after RANKL stimulation; D3, 3 days after RANKL stimulation; D5, 5 days after RANKL stimulation). Interestingly, in addition to circFam190a, the expression of many other circRNAs was also significantly altered during osteogenesis [Supplementary Fig. 1b]. These findings are consistent with previous sequencing reports by other researchers^55–58^, reinforcing the importance of circRNAs in osteoclast differentiation and providing potential targets for future studies on circRNAs during osteoclast differentiation.

Osteoporosis, characterized by increased bone resorption without compensatory bone formation, is a major health concern due to its severity, chronicity, and progression^59–63^. Current antiresorptive agents for treating osteoporosis, such as bisphosphonates and denosumab, are effective but far from ideal^64,65^. In our study, we show that depletion of circFam190a efficiently inhibits bone resorption and protects mice from OVX-induced osteoporosis [Figs. 2 and 3]. Intriguingly, neither up- nor downregulation of circFam190a affected bone formation [Fig. 3i and Supplementary Fig. 3b–h]. This differential effect of circFam190a on osteoclasts and osteoblasts can be attributed to the tissue- and stage-specific expression of circRNAs, which play various roles in different tissues^37,52^. Moreover, considering the high homology between mouse circFam190a and human circFAM190A [Supplementary Fig. 1g], as well as the significant elevation of circFAM190A in postmenopausal osteoporosis patients [Supplementary Fig. 1l], the unique characteristic of promoting bone resorption without affecting bone formation makes circFAM190A a promising target for treating osteoporosis.

ASOs are short synthetic single-stranded DNA/RNA oligonucleotides that selectively bind target RNA and regulate RNA function^66,67^. LNA ASOs, the third generation of ASOs with improved bioavailability and higher target RNA specificity, have shown promising results in clinical trials for diseases such as hepatitis and dyslipidemia^67,68^. However, the use of LNA-ASOs for treating osteoporosis and their efficacy and safety in bone disease remain unclear. Herein, we successfully utilized LNA-ASOs to knock down circRNAs both in vivo and in vitro [Supplementary Fig. 1d and Figs. 2a and 3a]. Moreover, treatment with LNA-ASOs targeting circFam190a effectively prevented OVX-induced osteoporosis. These findings provide evidence that LNA-ASOs could be a potential therapeutic approach for bone disease, offering a new avenue for the treatment of osteoporosis.

AKT1 has been well documented as a crucial regulator of osteoclast differentiation and survival^13,16^. Hsp90β, a ubiquitously and abundantly expressed molecular chaperone, is required for the stability and functional maturation of numerous client proteins, including AKT^69^. The binding of HSP90 with AKT prevents proteasomal degradation of AKT and contributes to the functional stabilization of AKT signaling^42,48^. Consistent with previous reports, our experiments confirmed the interaction between Hsp90 and AKT1. Furthermore, our data demonstrate that circFam190a binds directly to HSP90β and AKT1, forming a HSP90β-AKT1-circFam190a ternary complex [Fig. 4 and Supplementary Fig. 4]. Depletion of circFam190a significantly reduced the association between HSP90β and AKT1, while overexpression of circFam190a enhanced their interaction [Fig. 5e–j]. With the knockdown of circFam190a, AKT1 is subjected to ubiquitination and proteasomal degradation [Fig. 5a–d]. These findings highlight the unique role of circFam190a in enhancing the HSP90β/AKT1 complex, expanding our understanding of the molecular mechanisms between HSP90β and AKT1.

In subsequent studies, we further investigated whether circFam190a exerts its function on osteoclasts via the HSP90β-AKT1-circFam190a ternary complex. Modulating both HSP90β and AKT1 abrogated the effect of circFam190a on osteoclasts, indicating that circFam190a regulates osteoclasts in an HSP90β- and AKT1-dependent manner [Fig. 6 and Supplementary Fig. 6]. Additionally, in vivo mouse experiments suggested that inhibiting AKT signaling with MK-2206 successfully rescued the bone resorption phenotype induced by circFam190a [Fig. 7]. These data confirm our hypothesis that circFam190a regulates osteoclast function by modulating AKT1. Interestingly, we found that although treatment with MK-2206, an AKT signaling inhibitor, largely reduced the excessive osteoclast activity and the osteoporotic phenotype caused by the overexpression of circFam190a, it also led to a decline in bone formation [Fig. 7 and Supplementary Fig. 7]. Given that MK-2206 restrains AKT signaling in osteoblasts as well, this inhibitory effect on bone formation is reasonable. Nevertheless, this inhibitory effect on bone formation was overshadowed by MK-2206’s significant reduction in excessive osteoclast bone resorption, resulting in an overall increase in bone mass in mice. Of note, when knocking down circFam190a during osteoclastogenesis, a decrease in RANK mRNA expression was also observed [Fig. 2f]. RANK is a crucial cell surface receptor for osteoclastogenesis that initiates RANKL signaling^70^. Hence, the RNAK/RANKL signaling pathway might also be involved in the regulatory effects of circFam190a on osteoclasts. This result will be further verified in our subsequent studies.

In conclusion, we report that circFam190a, a circular RNA that binds directly to HSP90β and AKT1, is an important positive regulator of osteoclastogenesis. Targeting circFam190a could efficiently ameliorate OVX-induced osteoporosis. These findings contribute to our understanding of circular RNAs and bone metabolism and provide potential therapeutic targets for treating osteoporosis.

## Limitations of the study

From a mechanistic standpoint, while FUS is a major driver of circFam190a biogenesis, it remains unclear how FUS is upregulated during osteoclastogenesis. Additionally, while the enhanced interaction between HSP90β and AKT1 has been implicated in the positive effects of circFam190a on osteoclasts, there may be other underlying mechanisms that warrant further investigation. Furthermore, while this paper underscores the vital role of circFam190a, it is essential to acknowledge that numerous other circRNAs may also play important roles in osteoclast differentiation, necessitating future evaluation.

### Supplementary information

Supplementary Data
