# Supplementary material for: CircFam190a: a critical positive regulator of osteoclast differentiation via enhancement of the AKT1/HSP90β complex
Source: Exp Mol Med. 2023 Sep 1;55(9):2051–66. doi: 10.1038/s12276-023-01085-y (PMC10545668; doi:10.1038/s12276-023-01085-y)
Supplement: Supplementary file 1 — Supplementary Data [file 12276_2023_1085_MOESM1_ESM.pdf]

Supplementary Figures

Supplementary Fig. 1

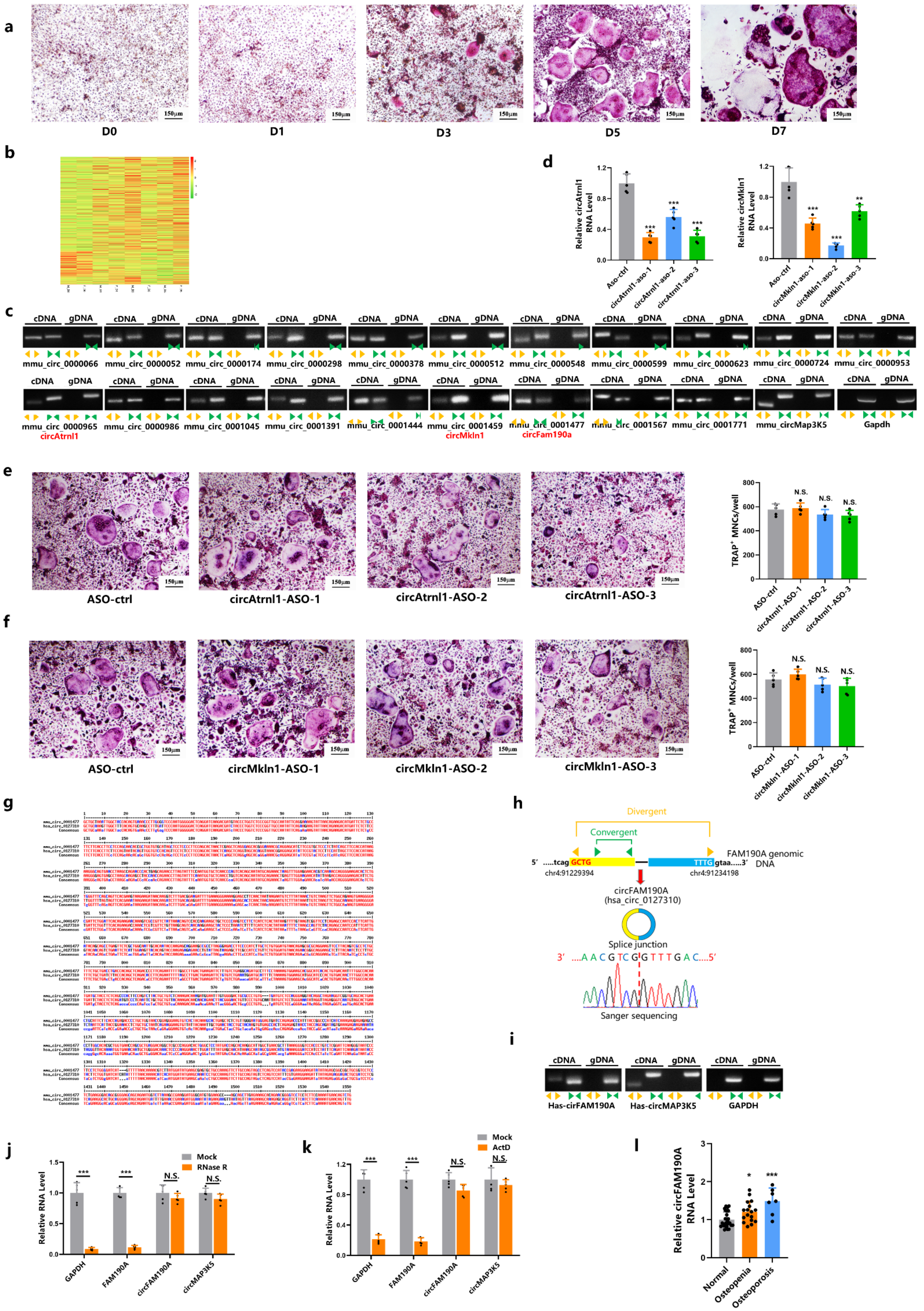

Supplementary Fig.1 | Supplementary Figure to Figure1 (a) TRAP staining of different osteoclast differentiation stages. BMMs were treated with M-CSF and RNAKL for indicated times. Data shown are representative of three independent experiments. **(b)** Heatmap of the circRNAs detected by circRNA-sequencing during osteoclastogenesis. **(c)** The existence of the indicated circRNAs was validated in osteoclasts by qRT-PCR. Divergent primers amplified circFam190a in cDNA but not genomic DNA (gDNA). circMap3k5 was used as a positive control, and Gapdh was used as a negative control. Yellow arrows indicate divergent primers, and green arrows indicate convergent primers. Data shown are representative of three independent experiments. **(d)** The relative RNA levels were measured by qRT-PCR. Statistical significance: ***$P < 0.001$ compared to Aso-ctrl group, **$P < 0.005$ compared to Aso-ctrl group, n=5. **(e)** TRAP staining and quantification of osteoclasts in control and circAtrnl1 knockdown groups. Statistical significance: N.S., not significant, n=5. **(f)** TRAP staining and quantification of osteoclasts in control and circMkln1 knockdown groups. Statistical significance: N.S., not significant, n=5. **(g)** The sequence alignment between mouse circFam190a and human circFAM190A was checked using the Multalin program. **(h)** Sequencing analysis of head-to-tail splicing junction in circFAM190A. **(i)** The existence of circFAM190a was validated in human PBMCs by qRT-PCR. Divergent primers amplified circFAM190A in

cDNA but not genomic DNA (gDNA). circMAP3k5 was used as a positive control, and GAPDH was used as a negative control. Yellow arrows indicate divergent primers, and green arrows indicate convergent primers. **(j)** The relative RNA levels were analyzed by qRT-PCR in human PBMCs treated with or without RNase R. Statistical significance: ***P < 0.001, N.S., not significant, n=5. **(k)** The relative RNA levels were measured by qRT-PCR in human PBMCs treated with or without ActD. Statistical significance: ***P < 0.001, N.S., not significant, n=5. **(l)** The relative circFAM190A RNA levels in PBMCs isolated from normal, osteopenia and osteoporosis patients were analyzed by qRT-PCR. Statistical significance: *P < 0.05 compared to Normal group, ***P < 0.001 compared to Normal group, n=26 in Normal group, n=17 in Osteopenia group and n=7 in Osteoporosis group.

Supplementary Fig. 2

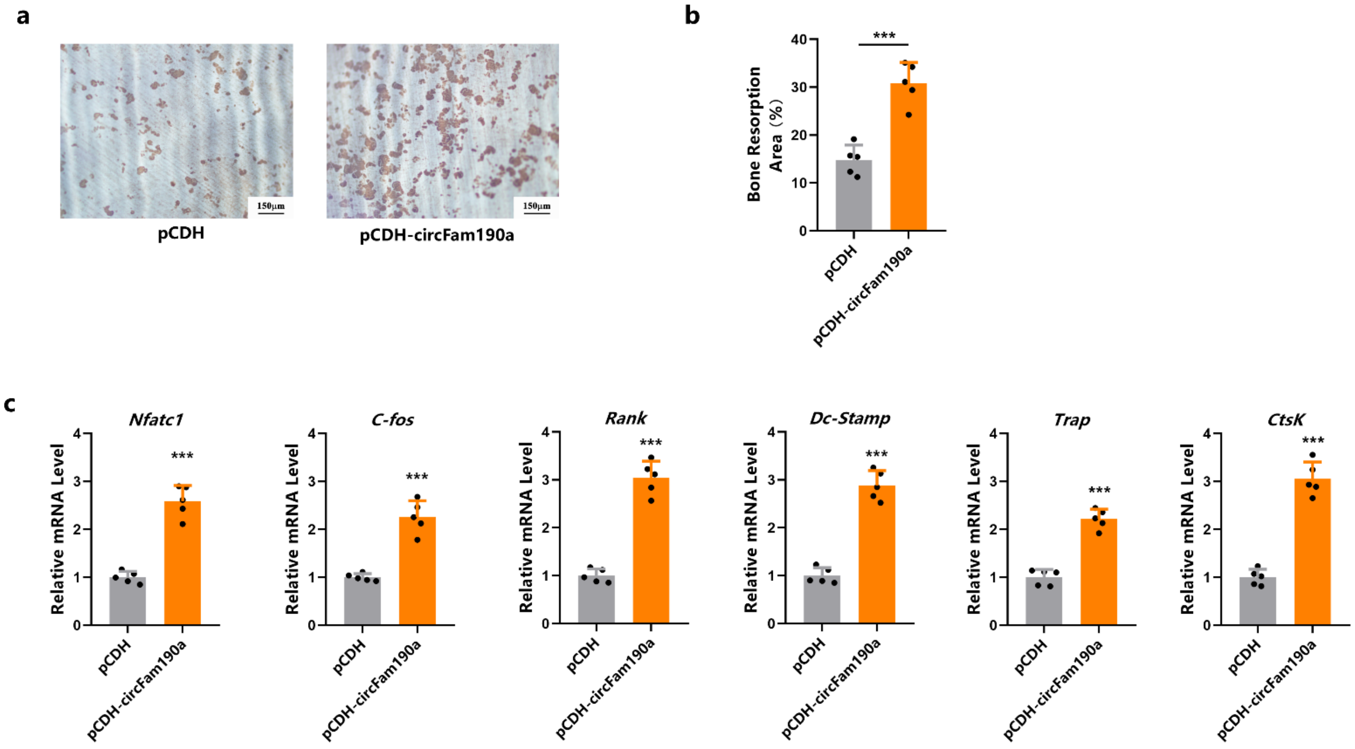

Supplementary Fig. 2 | Supplementary Figure to Fig. 2. (a and b) Bone resorption pit assay: representative images of dentin slice seeded with control or circFam190a overexpression osteoclasts (a) and quantification (b) of bone resorption area. The brown area indicates the bone resorption area. Statistical significance: ***P < 0.001, n=5. **(c)** The relative RNA levels of OC-specific genes in control and circFam190a overexpression osteoclasts. Statistical significance: ***P < 0.001, n=5.

Supplementary Fig. 3

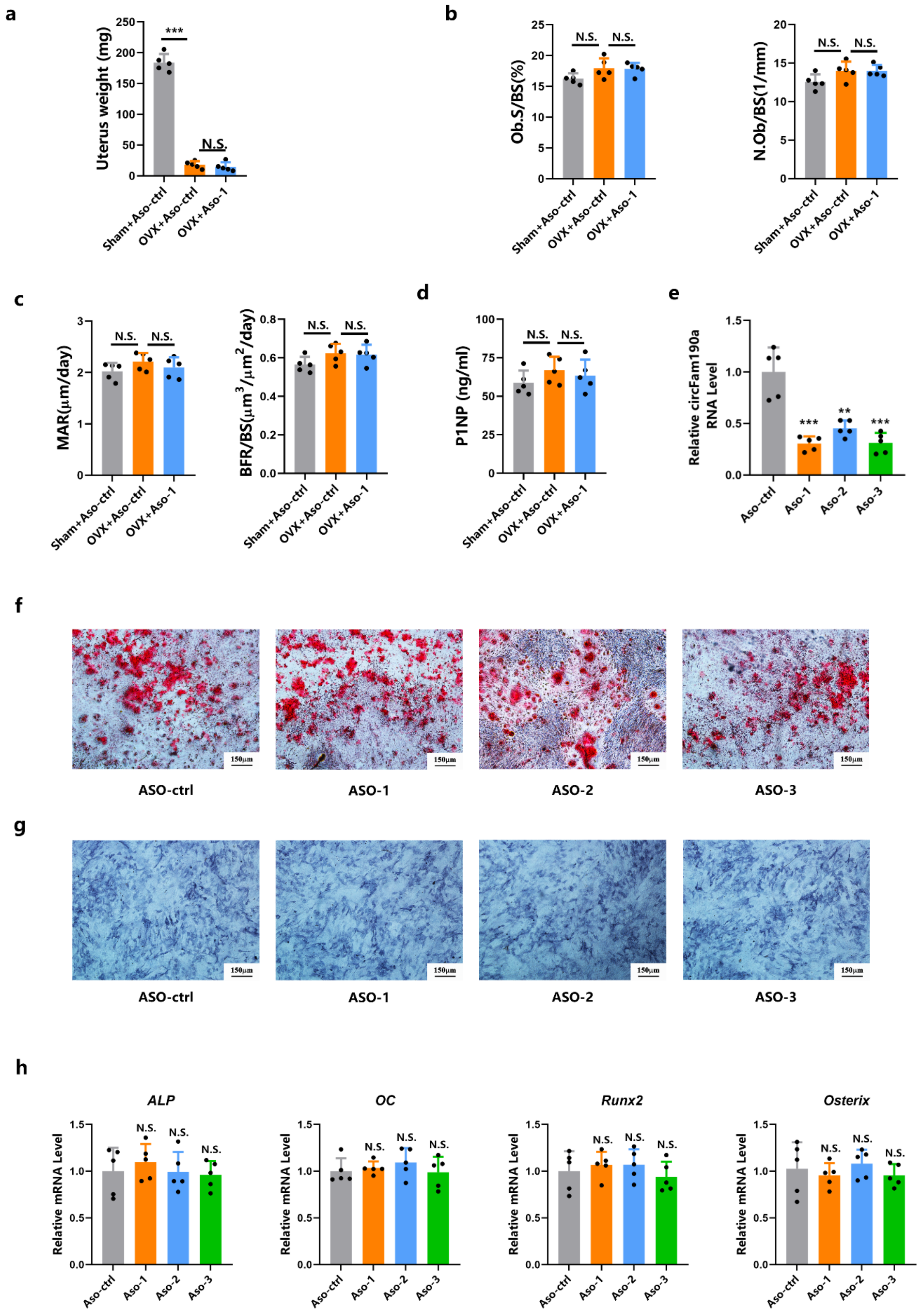

Supplementary Fig. 3 | Supplementary Figure to Fig. 3. (a) The uterus weight of mice from indicated groups. Statistical significance: ***P < 0.001 compared to "Sham + ASO-ctrl" group, n=5. **(b)** Histomorphometric analysis of osteoblast related parameters. Osteoblast Surface/Bone Surface: Ob.S/BS, Osteoblast Number/Bone Surface: Ob.N/BS, Statistical significance: N.S., not significant, n=5. **(c)** Histomorphometric analysis of bone formation related parameters. MAR: Mineral Apposition Rate, BFR/BS: Bone Formation Rate/Bone Surface, Statistical significance: N.S., not significant, n=5. **(d)** Serum bone formation marker P1NP levels from the indicated groups. Statistical significance: N.S., not significant, n=5. **(e)** Expression levels of circRNA circFam190a in BMSCs transfected with ASOs. Statistical significance: ***P < 0.001 compared to ASO-ctrl group, **P < 0.005 compared to ASO-ctrl group, n=5. **(f and g)** Alizarin Red staining (f) and ALP staining (g) of osteoblasts in the control and circFam190a knockdown groups. Data shown are representative of five independent experiments. **(h)** Relative RNA levels of osteoblast-specific genes in control and circFam190a knockdown osteoclasts in Day 14. Statistical significance: N.S., not significant, n=5.

Supplementary Fig. 4

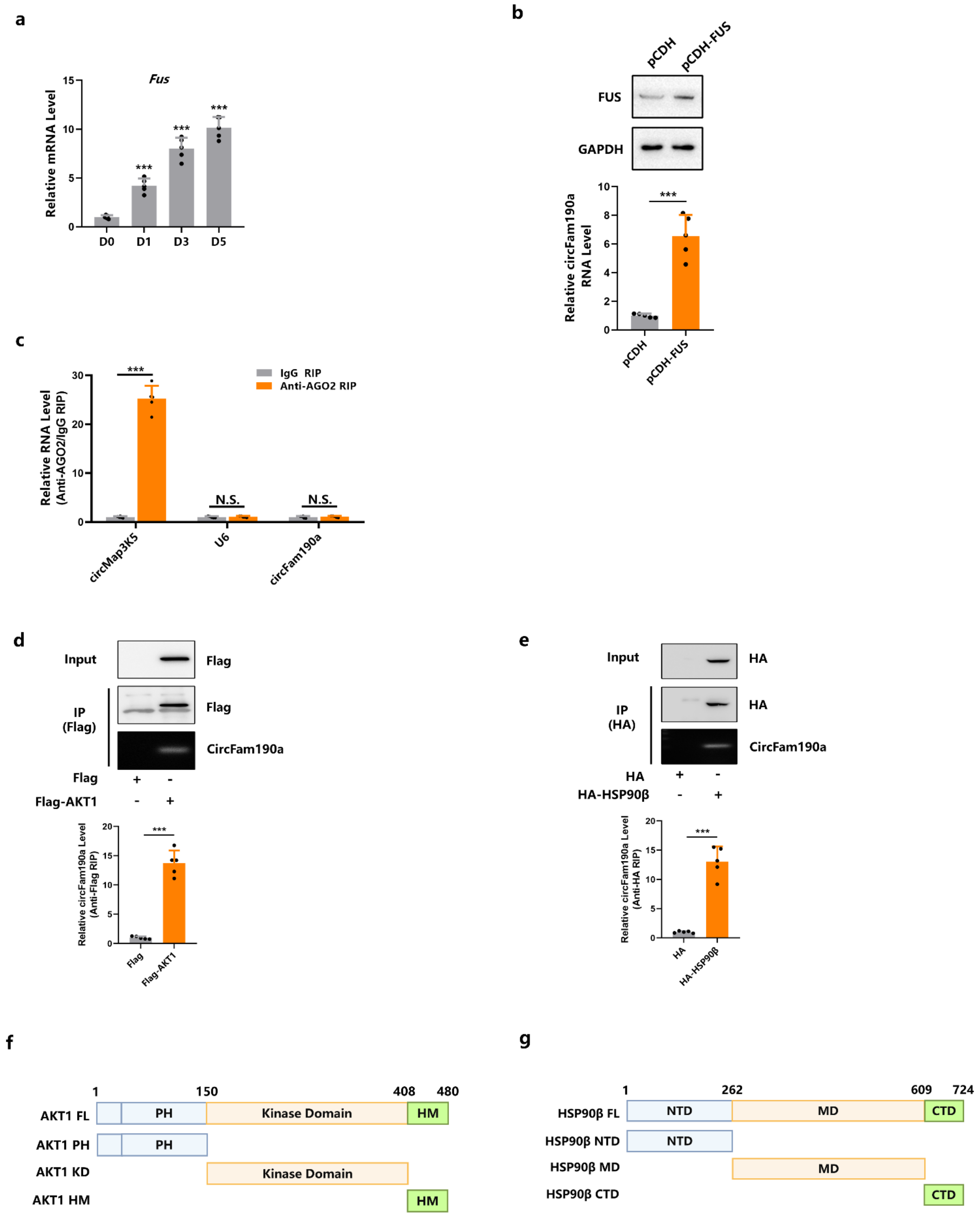

Supplementary Fig.4 | Supplementary Figure to Fig. 4. (a) The relative Fus mRNA levels in BMMs treated with RNAKL for indicated

times was detected by qRT-PCR. Statistical significance: ***P < 0.001, n=5. **(b)** The relative circFam190a RNA levels in osteoclasts with or without FUS overexpression were detected by qRT-PCR. Statistical significance: ***P < 0.001, n=5. **(c)** RIP analysis was carried out using anti-AGO2 or IgG antibodies. circFam190a, circMap3K5, and U6 levels in the samples were quantified using qRT-PCR. CircMap3K5 and U6 were applied as positive and negative controls that interacting with AGO2, respectively. Statistical significance: ***P < 0.001, N.S., not significant, n=5. **(d)** RIP assays in osteoclasts transfected with Flag or Flag-AKT1 using Flag antibodies. The precipitate was subjected to WB with the antibodies against Flag. The relative circFam190a levels were calculated by qRT-PCR. Statistical significance: ***P < 0.001, n=5 **(e)** RIP assays in osteoclasts transfected with HA or HA-HSP90β using HA antibody. The precipitate was subjected to WB with the antibodies against HA. The relative circFam190a levels were calculated by qRT-PCR. Statistical significance: ***P < 0.001, n=5. **(f)** Schematic illustration of AKT1 functional domains and corresponding truncation constructs. "PH" refers to "Pleckstrin Homology" and "HM" refers to "Hydrophobic Motif". **(g)** Schematic illustration of HSP90β functional domains and corresponding truncation constructs. "NTD" refers to "N-terminal domain", "MD" refers to "Middle Domain" and "CTD" refers to "C-terminal domain".

Supplementary Fig. 5

a

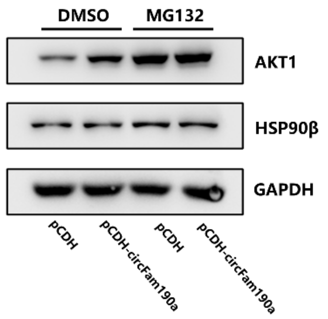

Supplementary Fig. 5 | Supplementary Figure to Fig. 5. (a) BMMs were transfected with either a control (pCDH) or circFam190a overexpression pCDH-circFam190a virus were treated with DMSO or 10 μM MG132 for 12 h. Whole cell lysates were collected for WB. Data shown are representative of three independent experiments.

Supplementary Fig. 6

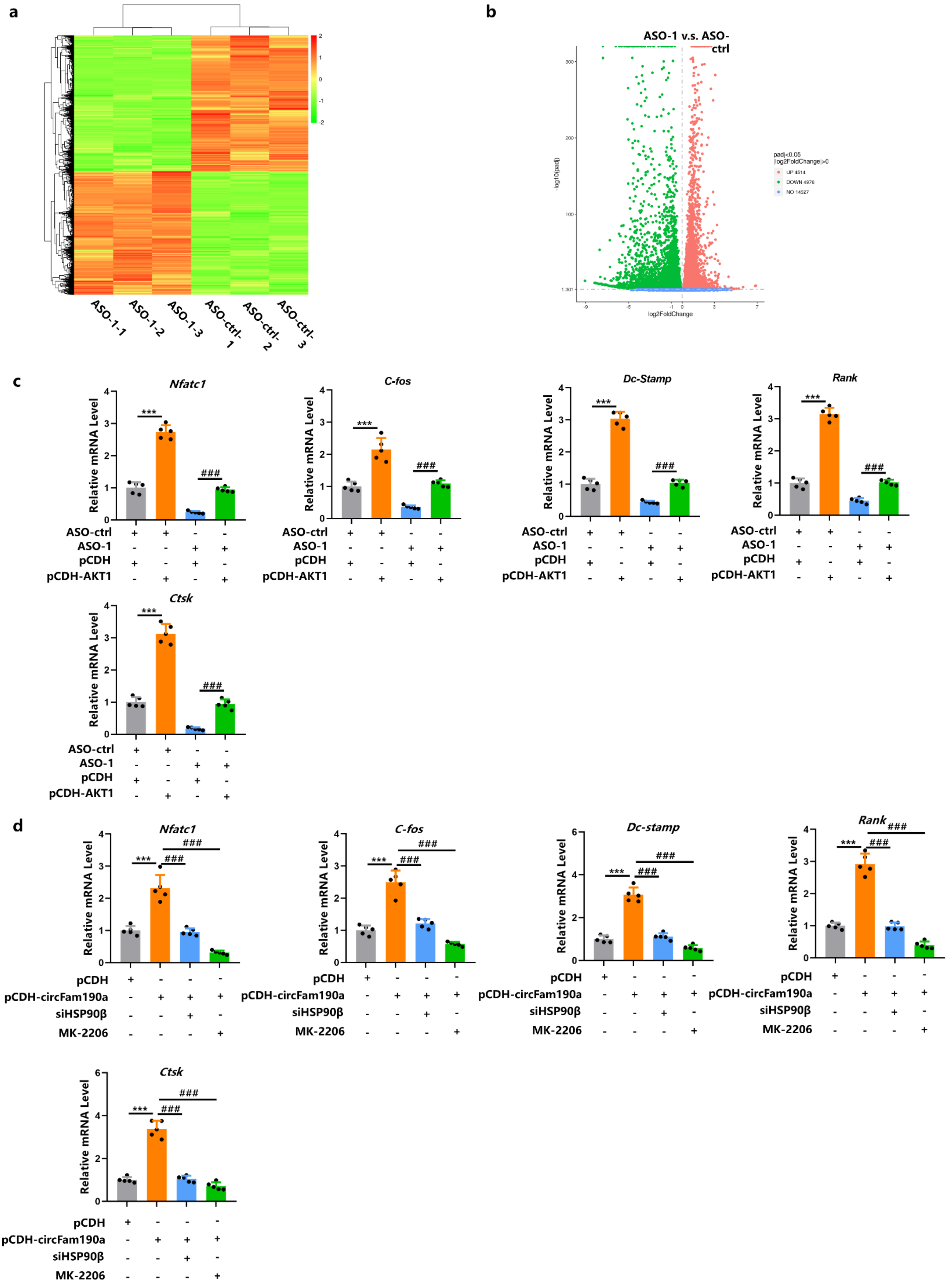

Supplementary Fig.6 | Supplementary Figure to Fig. 6. (a and b) Heatmap (a) and Volcano Plot (b) showing differential gene expression analysis based on mRNA sequencing data from osteoclasts with or without circFam190a knockdown. BMMs were transfected with control (Aso-Ctrl) or circFam190a knockdown (Aso-1) LNA-ASO and cultured with M-CSF and RANKL for 72 hours to induce osteoclast differentiation. Whole RNA samples were collected and subjected to mRNA sequencing. **(c)** Quantification of relative RNA levels of osteoclast-related genes in osteoclasts with or without circFam190a knockdown and AKT1 overexpression. BMMs were transfected with indicated ASO (ASO-ctrl or ASO-1) and virus (pCDH or pCDH-AKT1), and cultured with M-CSF and RANKL for 72 hours to induce osteoclasts differentiation. Statistical significance: ***P < 0.001 compared to "ASO-ctrl + pCDH" group, ###P < 0.001 compared to "ASO-1 + pCDH" group, n=5. **(d)** Quantification of relative RNA levels of osteoclast-related genes in osteoclasts with or without circFam190a overexpression and indicated treatment. BMMs were received indicated treatment (pCDH, pCDH-circFam190a or/and siHSP90β) and cultured with or without AKT inhibitor MK-2206 in the presence of M-CSF and RANKL for 72 hours to induce osteoclasts differentiation. Statistical significance: ***P < 0.001 compared to "pCDH" group, ###P < 0.001 compared to "pCDH-circFam190a" group, n=5.

Supplementary Fig. 7

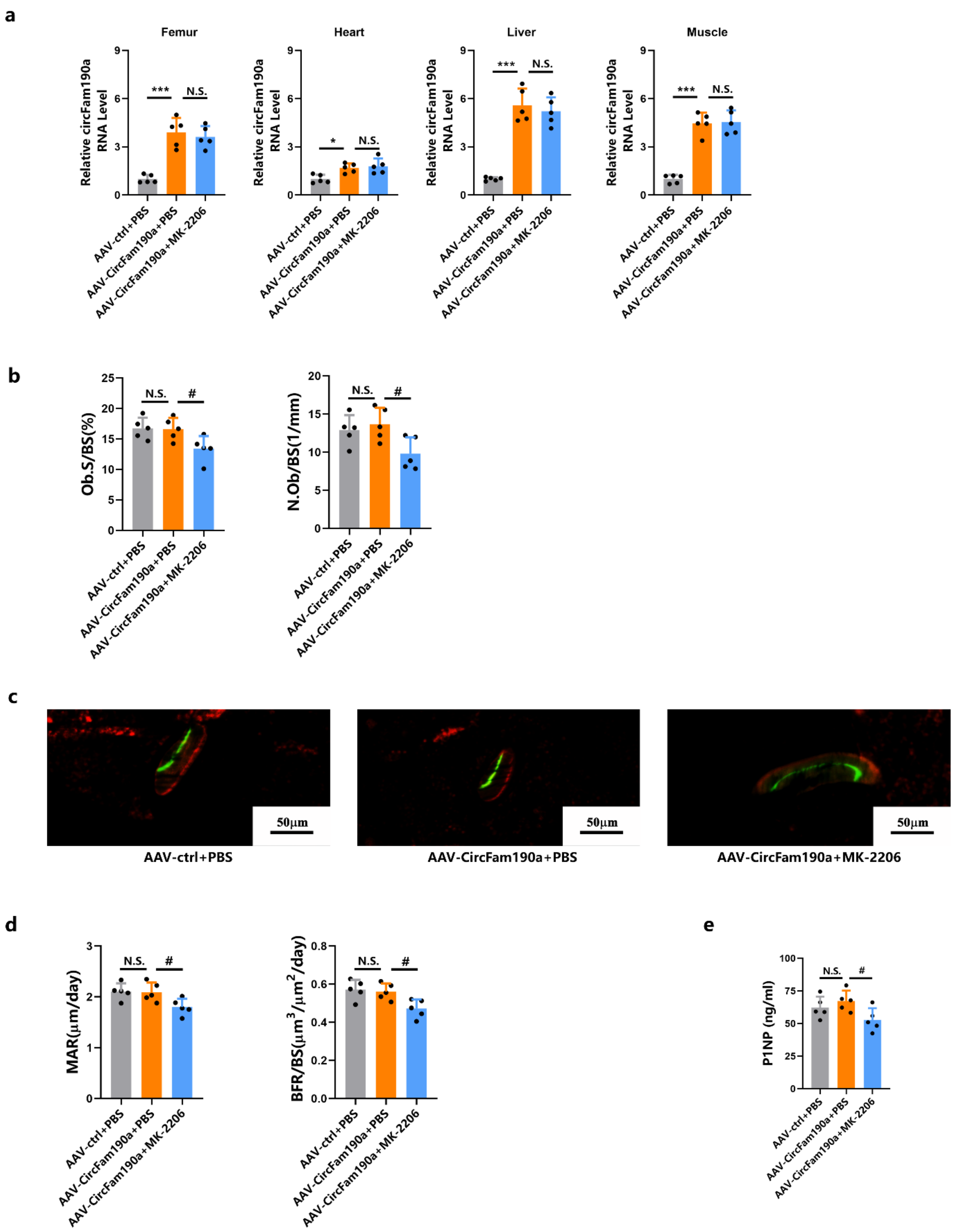

Supplementary Fig. 7 | Supplementary Figure to Fig. 7. (a)

Histomorphometric analysis of osteoblast related parameters, including Osteoblast Surface/Bone Surface (Ob.S/BS) and Osteoblast

Number/Bone Surface (Ob.N/BS). Statistical significance: #P < 0.05 compared to “AAV-circFam190a+PBS” group, N.S., not significant, n=5. **(b)** Representative double labelling images of trabecular bone from mice right femur. Green line indicates the first injection of Calcine and red line indicates the second injection of Alizarin red. **(c)** Histomorphometric analysis of bone formation related parameters. MAR: Mineral Apposition Rate, BFR/BS: Bone Formation Rate/Bone Surface. Statistical significance: #P < 0.05 compared to “AAV-circFam190a+PBS” group, N.S., not significant, n=5. **(d)** Serum P1NP levels from indicated mice. Statistical significance: #P < 0.05 compared to “AAV-circFam190a+PBS” group, N.S., not significant, n=5.

Supplementary Tables

Supplementary Table 1 Primers

Oligonucleotides for FISH and RNA pull-down	
**Oligonucleotide**	**Sequence (5'-3')**
circFam190a FISH	CACTGTGGTAGCCAATTTAGCAGCCAGACTGTTCATTTTGGAAGAGGAGG
circFam190a Sense	CTCCTCTTCCAAAATGAACAGTCTGGCTGCTAAATTGGCTACCACAGT
circFam190a Antisense	ACTGTGGTAGCCAATTTAGCAGCCAGACTGTTCATTTTGGAAGAGGAG

Primers used in qRT-PCR & Semi-quantitative RT-PCR analysis		
Gene	Primer	Sequence (5'-3')
mouse circFam190a Divergent (mmu_circ_0001477)	Forward	CTTAAAGCCGAAGGATGGGC
	Reverse	AGCCAATTTAGCAGCCAGACT
mouse circFam190a Convergent (mmu_circ_0001477)	Forward	TCCAATGGTGCTCAACCCAG
	Reverse	CGTGAACTGCTGAAACCCAC
mouse circAtrnl1 Divergent (mmu_circ_0000965)	Forward	ACCAGGACTTGGCCCAGATT
	Reverse	ACGGAAGGGCTGAAGGGTTTA
mouse circAtrnl1 Convergent (mmu_circ_0000965)	Forward	TCTCTGAATGTCCCTTCTACTGA
	Reverse	CTGCTTTATGTGAGGCCCGA
mouse circMkln1 Divergent (mmu_circ_0001459)	Forward	GCATGACAAATTGGTGTTAAAGGG
	Reverse	CGATAGCAGGCCTTTCAAGC
mouse circMkln1 Convergent (mmu_circ_0001459)	Forward	CAGGAAGCCATTCGCCTTTG
	Reverse	GGATGTTCCAGTGCAATCTTGG
mmu_circ_0000052 Divergent	Forward	CCCCAGTGATGCAAATAAGCC
	Reverse	ACAGGAGAGGCTTGTTCTTGAG
mmu_circ_0000052 Convergent	Forward	CAAGGGGCACCCCTACAAA
	Reverse	TCAGCTTATGGAGTACACGCT
mmu_circ_0000066 Divergent	Forward	AGTCTGCTGACCTTGAGCAC
	Reverse	CAGCAGTCCATTCTTGTAATTGCT
mmu_circ_0000066 Convergent	Forward	ACCAGCTGCTCTTCAGACAC
	Reverse	GGTGGCCTCTTTGTTTGCTG
mmu_circ_0000174 Divergent	Forward	GTGATGCAAATAAGCCCCTTGG
	Reverse	AGGCTTGTTCTTGAGACGTTG
mmu_circ_0000174 Convergent	Forward	TCATTGGACGGTGGCTTCTC
	Reverse	AGTGAACTCCAGTATGTCGGC
mmu_circ_0000298 Divergent	Forward	GCGGAACCCTACCCAGTTTC
	Reverse	TACACCGGTGACACATCCAC

mmu_circ_0000298 Convergent	Forward	CCATGTCAATGGACTGGTGGA
	Reverse	ATGGGCAATGGCCTTGAGTT
mmu_circ_0000378 Divergent	Forward	GGAGCCCCAAGAGCTTTAAGT
	Reverse	TGAGACGCAATGAGCTCAAGA
mmu_circ_0000378 Convergent	Forward	GAAGGAGCTGCAGAGGCAAAT
	Reverse	CTCCACAGACACATCAGTAGCC
mmu_circ_0000512 Divergent	Forward	CCCAACAAGGGTTTGACCTG
	Reverse	TGCTGTCTGAGTCCAGCAGA
mmu_circ_0000512 Convergent	Forward	CTTACAGAACCCCGCCAACTT
	Reverse	CATGAGGCTCTGCTCAAAGGG
mmu_circ_0000548 Divergent	Forward	CCTCATGGAAACACAAGAGCAG
	Reverse	AGACTCACAGAGACGAGACAC
mmu_circ_0000548 Convergent	Forward	ATGCCGCCCTGGATCAAATC
	Reverse	TAGGGCAAGCCATAGAACTGC
mmu_circ_0000599 Divergent	Forward	TCTTCACGGCCTGCTTTACTA
	Reverse	GTTTGACCCTCGCCTTCACTA
mmu_circ_0000599 Convergent	Forward	ACAGATCAGAGTGATGTTGCAGA
	Reverse	ATTTGCAAGTCCTTCTTACCGC
mmu_circ_0000623 Divergent	Forward	AACATGGTACCCAATGCGCT
	Reverse	GCATAGATTTGGAATCAGAACCAT
mmu_circ_0000623 Convergent	Forward	ATCCTAGCTGGGAAGAGGAGG
	Reverse	TTGTCCCCAGCTTGCTGAGTT
mmu_circ_0000724 Divergent	Forward	ACTTGGGCTAGAAGACTAGCAGTG
	Reverse	TCATGGGCCAGTGAGTGCAG
mmu_circ_0000724 Convergent	Forward	AGACCAGTCAGAGTGAGCCAA
	Reverse	GCGCTGCTGCTTAAGAAAGATG
mmu_circ_0000953 Divergent	Forward	AGTAGTGCTGTGGAGGAAAGC
	Reverse	GGCACCACTTCAATCTCTAGT
mmu_circ_0000953 Convergent	Forward	GGGAACAGCTCTCCTCAAGT
	Reverse	TCTTCTGGGGAAGACAACCT
mmu_circ_0000986 Divergent	Forward	ACACAGAAAAGACAGGTATGATGA
	Reverse	TGCTCATTTTTCTCACTCGCC
mmu_circ_0000986 Convergent	Forward	ACCAGAATTTCCTGTGTATATGCCT
	Reverse	CAGCCAGTCATCCATTTGCTC
mmu_circ_0001045 Divergent	Forward	GAGGAAAGCCTTAAGGAACTACTTT
	Reverse	TCTGATCAACTGGTAATTTCAACCA
mmu_circ_0001045 Convergent	Forward	TGAAAAGAGTCCAGAATGGTCCAA
	Reverse	GACAAGTGCAGCTTCTAGTCCT
mmu_circ_0001391 Divergent	Forward	GAAGATGAAGCAGGGGGAGAC
	Reverse	CCACGAAGGCTTTCACAGATT
mmu_circ_0001391 Convergent	Forward	TGCCATGAAGTGCTTAGACA
	Reverse	AGTGCTAACGAGAGACAGCA
mmu_circ_0001444 Divergent	Forward	CGGAGACTTGGAAGGACCAG
	Reverse	CGATTATATTCCGCACCCCT
mmu_circ_0001444 Convergent	Forward	GCGTACATTCTCTTGGCGGA
	Reverse	GCCGGTAACAACCATCTCCA
mmu_circ_0001567	Forward	GTCTTCCTGTGGAAAAGGTGGA

Divergent	Reverse	GCGCTTTCACTGAGTCCATTG
mmu_circ_0001567 Convergent	Forward	GTTCTGGATGTTGGGTGTGGA
	Reverse	AGGATTTCAGACTGGTCGACTG
mmu_circ_0001771 Divergent	Forward	GGTCACTGGAAACCTGCAGAC
	Reverse	ATGGTTGCCACTTCTCCTTCAA
mmu_circ_0001771 Convergent	Forward	GAAGGACAGCAGGTTTCAGC
	Reverse	CGTGTAGAGCTGGCAGAAGT
mouse circMap3k5 Divergent	Forward	TGAGCCAACACTACAGTCAG
	Reverse	GTTCCCGGTGCACACCAACT
mouse circMap3k5 Convergent	Forward	GGTCATTCAGGCATCCGAGAAG
	Reverse	CAGAAGTCCACGAGTTCCTGCT
mouse Gapdh Divergent	Forward	GGAGCCCTCCCTACTCTCTTG
	Reverse	CCAATACGGCCAAATCCGTT
mouse Gapdh Convergent	Forward	GTGATGGGTGTGAACCACGAG
	Reverse	GGTCATGAGCCCTTCCACAAT
human circFAM190A Divergent	Forward	GCAAAGAGCAGGTTCTTCATCT
	Reverse	CGTCTTGATCCTGAGTCCCC
human circFAM190A Convergent	Forward	CACAGGTAAACGGAGGAGCAT
	Reverse	GGTTCTGGTTGGTAGGCTCT
human circMAP3K5 Divergent	Forward	GAGCTCCGGAAAGTTGATGTG
	Reverse	TCGGTTGCATGAGCTCTGTC
human circMAP3K5 Convergent	Forward	AGAGGCTTGCTGGCATAAACCC
	Reverse	GCTGCTTTTCCGTAGCCTCTTG
human GAPDH Divergent	Forward	GAAGGTGAAGGTCGAGTC
	Reverse	GAAGATGGTGATGGGATTTC
human GAPDH Convergent	Forward	CAATGACCCCTTCATTGACC
	Reverse	TTGATTTTGGAGGGATCTCG
mouse Fam190a	Forward	TGTGAGCCCTTCCAGCAGTA
	Reverse	AGCCTCAGCATCGAGCATTCA
mouse pre-Fam190a	Forward	TTACTGACCACAAGCCAGCCTC
	Reverse	GTGGAGCAGGAGATGGGAAAGT
human FAM190A	Forward	CACAGGTAAACGGAGGAGCAT
	Reverse	GGTTCTGGTTGGTAGGCTCT
mouse U6	Forward	CTCGCTTCGGCAGCACA
	Reverse	AACGCTTCACGAATTTGCGT
mouse Fus	Forward	GCTTCAAACGACTATACCCAACA
	Reverse	GGCCATAACCACTGTAACTCTGT
mouse Nfatc1	Forward	GGAGAGTCCGAGAATCGAGAT
	Reverse	TTGCAGCTAGGAAGTACGTCT
mouse C-fos	Forward	CGGGTTTCAACGCCGACTA
	Reverse	TTGGCACTAGAGGACAGA
mouse Rank	Forward	TCCACTTAGACTACTGCAAGCA
	Reverse	CATCTTCGGCGTTTACTACAGG
mouse Dc-stamp	Forward	AAAACCCTTGGGCTGTTCTT
	Reverse	CTTCGCATGCAGGTATTCAA
mouse Trap	Forward	TCCTGGCTCAAAAAGCAGTT
	Reverse	ACATAGCCCACACCGTTCTC

mouse Ctsk	Forward	CAGCTTCCCCAAGATGTGAT
	Reverse	AAAAATGCCCTGTTGTGTCC
**Locked Nucleic Acid enhanced Antisense Oligonucleotides (LNA-ASO) for circular RNA knock down**		
**Oligonucleotide**	**Target**	**Sequence (5'-3')**
ASO-1	circFam190a	CAATTTAGCAGCCAGACTG
ASO-2	circFam190a	GCAGCCAGACTGTTCA
ASO-3	circFam190a	CCAATTTAGCAGCCAGAC
circAtrnl1-ASO-1	circAtrnl1	CAATCTGGGCCAAGTCCTG
circAtrnl1-ASO-2	circAtrnl1	AACAATCTGGGCCAAGT
circAtrnl1-ASO-3	circAtrnl1	TGGGCCAAGTCCTGGTG
circMkln1-ASO-1	circMkln1	GAATCAAGTACATTTACAG
circMkln1-ASO-2	circMkln1	AGAATCAAGTACATTTAC
circMkln1-ASO-3	circMkln1	TCAGAATCAAGTACATT
ASO-ctrl	Negative Control	TCATACTATATGACAG
**small interfering RNAs (siRNAs) for gene knock down**		
**Oligonucleotide**	**Target**	**Sequence (5'-3')**
siFUS	Fus	GTCCTAATCCTACATGTGAGA
siHSP84	Hsp84	GAGCTGATACCTGAGTACCTCAACT
si-Ctrl	Negative Control	GAGAGATCCGTGAATCCCTATCACT

Supplementary Table 2 Patients Information

No.	Name	Patient ID	Gender	Age	Date of Birth	T Value	Group
1	XXX	22200043	Female	51	1971/5/28	-2	Osteopenia
2	XXX	22201485	Female	51	1971/7/5	-1.9	Osteopenia
3	XXX	22204477	Female	51	1970/9/10	1.5	Normal
4	XXX	22205637	Female	51	1971/8/14	1.303	Normal
5	XXX	22208379	Female	51	1970/9/15	2.4	Normal
6	XXX	22117496	Female	52	1969/11/8	1.195	Normal
7	XXX	22096359	Female	52	1970/4/16	1.014	Normal
8	XXX	22200105	Female	52	1969/8/25	0	Normal
9	XXX	22203772	Female	52	1969/12/1	-0.6	Normal
10	XXX	22199267	Female	52	1970/7/14	-1.1	Osteopenia
11	XXX	22194370	Female	53	1968/10/20	0.5	Normal
12	XXX	22197677	Female	53	1968/10/11	1.8	Normal
13	XXX	22201478	Female	53	1969/1/18	-0.1	Normal
14	XXX	22201891	Female	53	1969/6/26	-1.3	Osteopenia
15	XXX	22204628	Female	53	1969/4/30	0.2	Normal
16	XXX	22091953	Female	54	1968/6/20	-1.6	Osteopenia
17	XXX	22201518	Female	54	1967/10/21	-0.1	Normal
18	XXX	22201512	Female	54	1968/2/17	-2.4	Osteopenia
19	XXX	22210271	Female	54	1967/9/8	-2.6	Osteoporosis
20	XXX	22213201	Female	54	1968/8/1	0.2	Normal
21	XXX	22191133	Female	55	1967/1/26	-1.4	Osteopenia
22	XXX	22192177	Female	55	1967/8/8	-0.3	Normal
23	XXX	22201513	Female	55	1967/8/16	-0.3	Normal
24	XXX	22096106	Female	55	1967/4/15	-1	Osteopenia
25	XXX	22204465	Female	55	1967/3/10	-0.3	Normal
26	XXX	22194466	Female	56	1965/10/19	-0.4	Normal
27	XXX	22201487	Female	56	1965/10/9	-2.5	Osteoporosis
28	XXX	22203635	Female	56	1966/8/1	-2.5	Osteoporosis
29	XXX	22204597	Female	56	1966/5/5	-1.2	Osteopenia
30	XXX	22205497	Female	56	1966/8/4	-1	Osteopenia
31	XXX	22191102	Female	57	1965/7/4	-1.7	Osteopenia
32	XXX	22194394	Female	57	1965/5/23	-0.8	Normal
33	XXX	22201484	Female	57	1965/8/9	2.3	Normal
34	XXX	22201524	Female	57	1965/5/20	-1	Osteopenia
35	XXX	22093675	Female	57	1965/7/29	-0.4	Normal
36	XXX	22190201	Female	58	1964/4/18	-2.6	Osteoporosis
37	XXX	22202755	Female	58	1964/2/1	1.4	Normal
38	XXX	22209467	Female	58	1963/9/21	-2.2	Osteopenia
39	XXX	22212937	Female	58	1964/5/9	-1	Osteopenia
40	XXX	22190206	Female	59	1963/3/14	-2.9	Osteoporosis
41	XXX	22191088	Female	59	1963/8/9	0.1	Normal
42	XXX	22194358	Female	59	1962/10/4	-1.4	Osteopenia
43	XXX	22197707	Female	59	1962/9/28	-2.8	Osteoporosis
44	XXX	22204510	Female	59	1963/7/13	0	Normal

45	XXX	22186118	Female	60	1962/4/20	-0.1	Normal
46	XXX	22141763	Female	60	1961/9/22	-2.3	Osteopenia
47	XXX	22212913	Female	60	1962/6/29	-0.1	Normal
48	XXX	22214384	Female	60	1962/8/1	-0.6	Normal
49	XXX	22186448	Female	60	1962/1/15	-1.8	Osteopenia
50	XXX	22229232	Female	63	1959/6/29	-3.4	Osteoporosis

Supplementary Table 3 Antibodies

Antibody	Source	IDENTIFIER
FUS	CST	67840S
AKT1	CST	2938
P-AKT1	CST	9018S
HSP84	CST	5087S
FLAG	CST	14793S
HA	CST	3724S
PDIA6	Proteintech	66669-1-Ig
GAPDH	Proteintech	60004-1-Ig
